# *N*-Substituted l-Iminosugars for the Treatment of Sanfilippo Type B Syndrome

**DOI:** 10.1021/acs.jmedchem.2c01617

**Published:** 2023-01-25

**Authors:** Valeria De Pasquale, Anna Esposito, Gianluca Scerra, Melania Scarcella, Mariangela Ciampa, Antonietta Luongo, Daniele D’Alonzo, Annalisa Guaragna, Massimo D’Agostino, Luigi Michele Pavone

**Affiliations:** † Department of Molecular Medicine and Medical Biotechnology, University of Naples Federico II, Via S. Pansini 5, 80131 Naples, Italy; ‡ Department of Veterinary Medicine and Animal Productions, University of Naples Federico II, Via F. Delpino 1, 80137 Naples, Italy; § Department of Chemical, Materials and Production Engineering, University of Naples Federico II, Piazzale V. Tecchio 80, 80125 Naples, Italy; ∥ AORN Sant’Anna e San Sebastiano, Via F. Palasciano, 81100 Caserta, Italy; ⊥ Department of Chemical Sciences, University of Naples Federico II, Via Cintia, 80126 Napoli, Italy

## Abstract

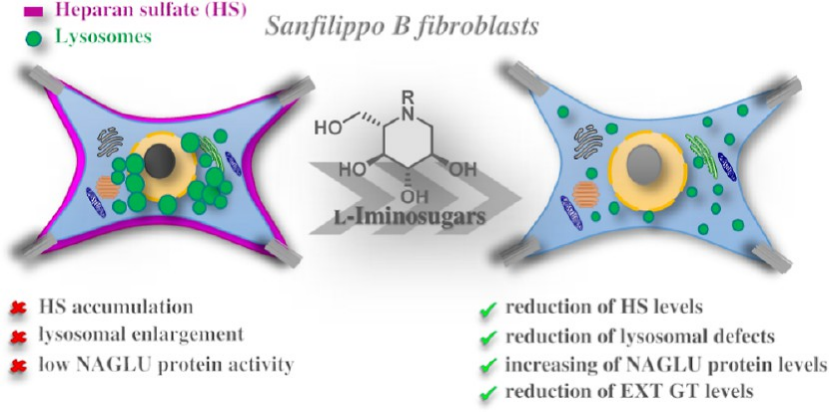

Sanfilippo syndrome comprises a group of four genetic diseases due to the lack or decreased activity of enzymes involved in heparan sulfate (HS) catabolism. HS accumulation in lysosomes and other cellular compartments results in tissue and organ dysfunctions, leading to a wide range of clinical symptoms including severe neurodegeneration. To date, no approved treatments for Sanfilippo disease exist. Here, we report the ability of *N*-substituted l-iminosugars to significantly reduce substrate storage and lysosomal dysfunctions in Sanfilippo fibroblasts and in a neuronal cellular model of Sanfilippo B subtype. Particularly, we found that they increase the levels of defective α-*N*-acetylglucosaminidase and correct its proper sorting toward the lysosomal compartment. Furthermore, l-iminosugars reduce HS accumulation by downregulating protein levels of exostosin glycosyltransferases. These results highlight an interesting pharmacological potential of these glycomimetics in Sanfilippo syndrome, paving the way for the development of novel therapeutic approaches for the treatment of such incurable disease.

## Introduction

Sanfilippo syndrome, or mucopolysaccharidosis (MPS) type III, is a lysosomal storage disease (LSD) consisting of four disease subtypes, namely Sanfilippo A, B, C, and D, all caused by deficiencies of enzymes involved in the catabolism of the glycosaminoglycan (GAG) heparan sulfate (HS).^1^ Defective enzymes are *N*-sulfoglucosamine sulfohydrolase (SGSH) (EC 3.10.1.1) for Sanfilippo A, *N*-acetyl-α-d-glucosaminidase (NAGLU) (EC 3.2.1.50) for Sanfilippo B, acetyl-CoA:α-glucosaminide *N*-acetyltransferase (HGSNAT) (EC 2.3.1.78) for Sanfilippo C, and *N*-acetylglucosamine-6-sulfate sulfatase (GNS) (EC 3.1.6.14) for Sanfilippo D. Although all Sanfilippo subtypes are rare diseases, Sanfilippo A and B are more common than C and D with the incidence of 0.29–1.89 and 0.42–0.72 per 100,000 births, respectively.^2,3^ In all Sanfilippo subtypes, as a result of enzymatic defects, undegraded HS accumulates in cellular substructures, like lysosomes and cell membrane, leading to a plethora of clinical manifestations, which involve various organs and tissues including skeletal muscles, heart, lungs, and especially the central nervous system (CNS).^4^ Indeed, the clinical course of patients affected by Sanfilippo disease is characterized by progressive CNS degeneration, leading to cognitive decline and autism spectrum disorders.^4^ Several efforts have been devoted to the identification of novel treatments for the Sanfilippo syndrome. To date, the main strategies involve (i) substrate reduction therapy (SRT), based on the administration of drugs that inhibit HS synthesis; (ii) enzyme replacement therapy (ERT), aiming to deliver the missing enzyme to target tissues; (iii) gene therapy (GT) that leverages viral vectors—such as adeno-associated vectors (AAVs) or lentiviral vectors (LVs)—in order to transfer the correct enzyme-coding genes, and (iv) hematopoietic stem cell transplantation (HSCT), which supplies the enzyme secreted by healthy donor cells to recipient patients.^5^ Although all the aforementioned therapies have demonstrated promising results in preclinical studies,^6−9^ clinical ones highlighted no-to-low effect on patients, especially for CNS impairment.^10−12^ Thus, the identification of novel therapeutic approaches for this intractable disease is still highly demanding.

**Figure 1:**
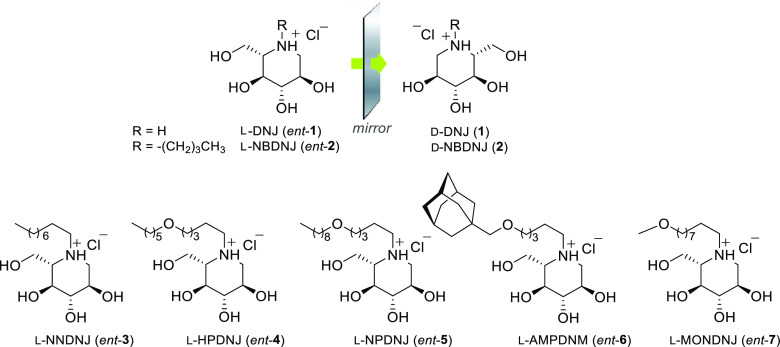
l-Iminosugars evaluated in Sanfilippo disease cellular models.

Over the last few decades, iminosugars, glycomimetics with an amino function replacing the endocyclic oxygen of natural carbohydrates, exhibited a notable pharmacological potential in the LSD treatment stewardship, thanks to their ability to interact with carbohydrate-processing enzymes and alter their properties.^13−16^ In particular, some iminosugars have found application in the treatment of LSDs either for their ability to inhibit substrate synthesis (SRT)^17,18^ and consequent lysosomal accumulation or to bind, reversibly and at sub-inhibitory concentrations, mutated lysosomal enzymes, thus enhancing or restoring their function (pharmacological chaperone therapy—PCT).^19,20^ To date, two iminosugars are commercially available for the treatment of LSDs: ZAVESCA (miglustat, also known as d-NBDNJ, *N*-butyl-d-deoxynojirimycin, compound **2**) (Figure 1), licensed within the SRT for the treatment of type I Gaucher’s disease^21,22^ and Niemann–Pick type C disease,^23^ and Galafold (migalastat, also known as DGJ, 1-deoxygalactonojirimycin), at present the only approved pharmacological chaperone for Fabry disease.^24,25^ In addition, many other iminosugars have been evaluated for their use as drug candidates in different LSDs, including MPSs.^26,27^ In this field, the activity of some iminosugar derivatives acting as pharmacological chaperones for the treatment of MPS II, III, and IV was evaluated.^28−31^ Furthermore, an interesting application of iminosugars in MPSs involves the assumption that inhibition of ganglioside secondary storage can represent a therapeutic strategy for patients with neurological involvement. On these bases, miglustat was evaluated as a substrate-reducing agent for Sanfilippo diseases due to its ability to interfere with glycosphingolipid metabolism.^32^ However, despite the promising results obtained in preclinical studies,^33^ no beneficial effects were observed in Sanfilippo patients treated with miglustat.^34^ Overall, these findings suggest that iminosugars represent attractive drug candidates for the treatment of Sanfilippo disease and therefore further efforts should be devoted to identify novel iminosugars effective in reducing HS and lysosomal accumulation in Sanfilippo patients. In the frame of our previous studies on the role of chirality in the pharmacological activity of iminosugars and other bioactive compounds,^35−38^ we recently highlighted the promising potential of unnatural l-*gluco*-configured iminosugars for the treatment of rare diseases. Particularly, l-NBDNJ (*ent*-**2**, Figure 1), the synthetic enantiomer of d-NBDNJ (**2**), showed an interesting potential for the treatment of Pompe lysosomal disease, while not acting as an inhibitor of the most common glycosidases, differently from its enantiomer.^39^ Even more interesting results have been obtained by us when l-iminosugars were evaluated in cystic fibrosis (CF).^40^ Indeed, *ent*-**2** and its *N*-substituted congeners exhibited strong anti-inflammatory and antibacterial properties *in vitro* and *in vivo*, pointing out the potential use of these compounds for the treatment of CF lung disease.^40,41^

Based on the established therapeutic activity of d-iminosugars in the treatment of LSDs, as well as on the promising biological properties exhibited by the unnatural l-iminosugars,^42−44^ the pharmacological potential of seven l-iminosugars was herein evaluated in Sanfilippo B disease. l-Deoxynojirimycin (**l-DNJ**, *ent*-**1**), its *N*-alkyl derivatives (*N*-butyl l-DNJ, **l-NBDNJ**, *ent*-**2**; *N*-nonyl l-DNJ, **l-NNDNJ**, *ent*-**3**), and *N*-alkoxyalkyl derivatives (*N*-hexyloxypentyl l-DNJ, **l-HPDNJ**, *ent*-**4**; *N*-nonyloxypentyl l-DNJ, **l-NPDNJ**, *ent*-**5**; *N*-adamantanemethoxypentyl l-DNJ, **l-AMPDNM**, *ent*-**6**; *N*-methoxynonyl l-DNJ, **l-MONDNJ**, *ent*-**7**) (Figure 1) were considered due to their interesting *in vitro* and *in vivo* activity toward other pathologies.^39−41^ In the present study, an alternative path for the synthesis of *ent*-**1**, whose subsequent *N*-alkylation led to *ent*-(**2**–**7**), was first described. Subsequently, the capability to reduce HS accumulation and to rescue lysosomal defects by *ent*-(**1**–**7**) was demonstrated in a Sanfilippo B neuronal cellular model generated in our laboratory^45^ and then confirmed in fibroblasts derived from Sanfilippo B patients. Furthermore, by using HeLa human epithelial cervical cancer cell line and Sanfilippo B fibroblasts, the molecular mechanism of action of the active l-iminosugars was investigated to assess whether the observed activity could be ascribed to their ability to inhibit a critical step of HS biosynthetic machinery or to increase NAGLU levels and activity.

## Results

### Chemistry

**Scheme 1:**
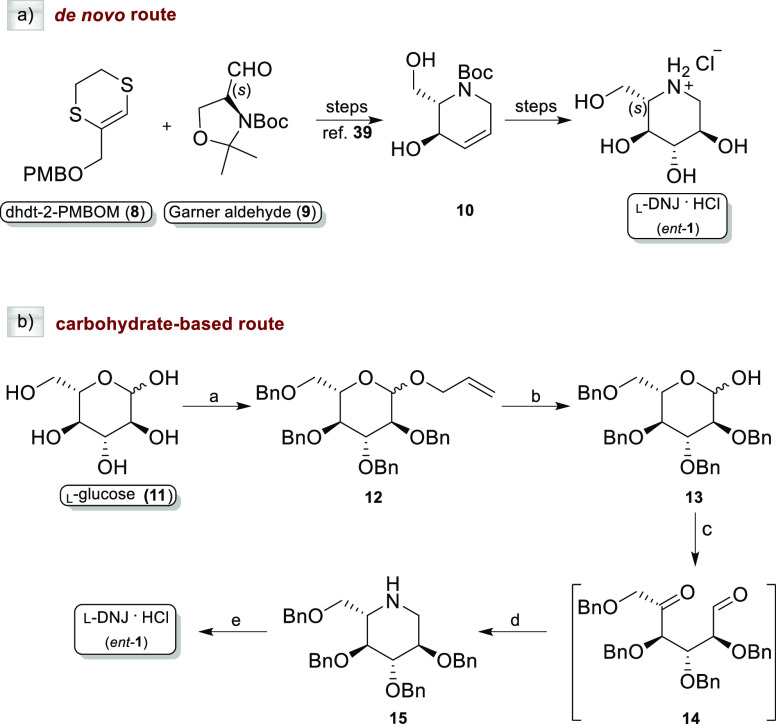
Synthesis of *ent*-1 by (a) *De Novo* Route and (b) Carbohydrate-Based Route Reagents and conditions: (a) (i): AllOH, BF_3_·OEt; Δ, on; (ii): BnBr, NaH, DMF, 0 °C, then rt, on, 91% overall yield; (b) PdCl_2_, MeOH, rt, on, 95% (c) (i): LiAlH_4_, THF, 0 °C, 20 h at rt; (ii): (COCl)_2_, DMSO, TEA, DCM; (d) NaBH_3_CN, NH_4_OAc, Na_2_SO_4_, MeOH, 75% over three steps; and (e) 1 M, BCl_3_, DCM, 0 °C, 12 h, 92%.

We recently carried out a highly stereocontrolled *de novo* synthesis of *ent*-**1** and *ent*-**2**,^39,40^ the unnatural enantiomers of d-DNJ (**1**) and d-NBDNJ (**2**), respectively, along with a series of *N*-substituted l-deoxyiminosugars (*ent*-**3-7**) bearing alkyl and alkoxy alkyl chains of different lengths and polarities. As previously reported by us,^39^ the synthesis of the iminosaccharide scaffold *ent*-**1** can be obtained starting from a heterocyclic homologating agent (dhdt-2-PMBOM, **8**) and l-Garner aldehyde (**9**) to fix the l-configuration at the first steps of the synthetic path (Scheme 1a). Alternatively, as described herein, *ent*-**1** can be synthesized from the commercially available l-glucose (**11**), which already has on the skeleton all the stereocenters with the required configuration. A synthetic procedure already reported^46^ for the preparation of the natural d-DNJ has been exploited herein with slight modifications (Scheme 1b). Briefly, starting from **11**, transient protection of the anomeric center (AllOH and BF_3_·OEt), followed by benzylation of the remaining OH groups (NaH and BnBr) and removal of the allyl group provided 2,3,4,6-tetra-*O*-benzyl-l-glucopyranose (**13**). Sequential LiAlH_4_-mediated reduction, Swern oxidation [(CO)_2_Cl_2_/DMSO, then Et_3_N], and reductive amination (NaBH_3_CN/AcONH_4_) of **13** gave the tetra-*O*-benzyl l-deoxynojirimycin (**15**) in 75% yield. Finally, de-*O*-benzylation was performed with 1 M BCl_3_ in DCM at 0 °C, to obtain the pure *ent*-**1** as the hydrochloride salt (92% yield).

The *N*-functionalization of *ent*-**1** to obtain its *N*-alkyl and *N*-alkoxy alkyl derivatives was performed by a synthetic protocol involving the use of the well-known polymer-supported triphenylphosphine (PS-TPP)/iodine system as reported by us earlier.^40^

**Scheme 2:**
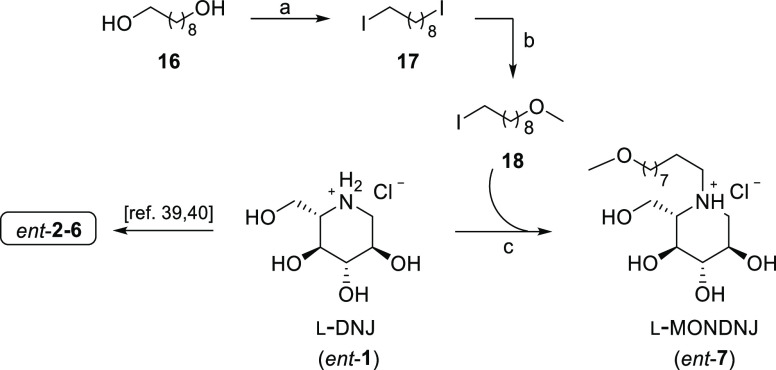
Synthesis of *ent*-(**2–7**) Reagents and conditions: (a) PS-TPP, I_2_, DCM, rt, 1 h, 95%; (b) MeOH, NaH, THF, 0 °C for 1 h then rt, 48 h, 75%; (c) (i): l-DNJ (*ent*-**1**), K_2_CO_3_, DMF, 80 °C, on, 75%; (ii): MeOH, HCl 1 M, rt, quantitative.

This procedure was herein exploited to prepare *ent*-**7**, whose synthesis has never been reported before (Scheme 2). Our route involved a PS-TTP/I_2_-mediated double iodination of 1,9-nonanediol (**16**) to provide 1,9-diiodononane (**17**).

The PS-TPP/I_2_ activating system (either in combination or without imidazole) has been already employed in many synthetic studies aimed to achieve different chemical transformations.^36,37,47^ In this case, it can be employed in the absence of imidazole, thus allowing us to devise a synthetic procedure not requiring chromatographic purification. As a matter of fact, the resin-bound phosphine oxide, representing the sole reaction byproduct, can be simply filtered off and in this case recycled by reduction to the starting phosphine form.^48^

Subsequent treatment of *bis*-iodide **17** with *in situ*-generated sodium methoxide afforded methoxy ether **18** in 75% yield. Reaction of **18** with *ent*-**1** under standard conditions (K_2_CO_3_) and treatment with HCl 1 M gave *ent*-**7** as a hydrochloride salt (75% yield). Our approach enables obtaining the desired alkylated iminosugars in only a few reaction steps in satisfying yields, limiting the extractive workup and chromatographic purification stages.

### Biological Evaluation

#### l-Iminosugars Reduce Lysosomal Defects and HS Accumulation in NAGLU-Silenced Neuroblastoma SK-NBE

**Figure 2:**
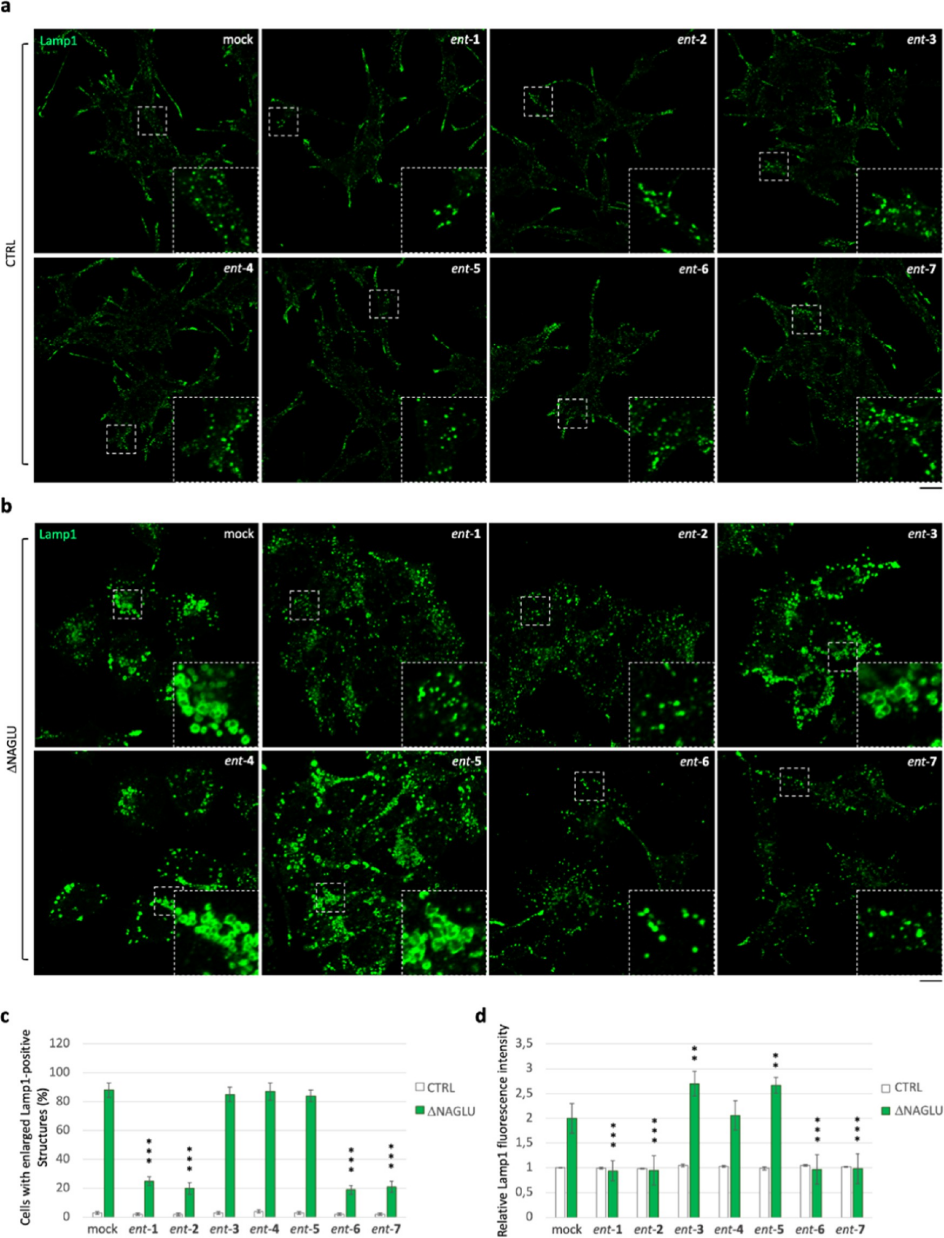
Activity of *ent*-(**1**–**7**) in rescuing the lysosomal phenotype in the Sanfilippo B cellular model. (a) Lysosomal staining of CTRL clone treated with *ent*-(**1**–**7**): control stable clone seeded on coverslips was grown for 48 h in the presence of 20 μM of each l-iminosugar and then processed for indirect immunofluorescence by using a specific antibody against Lamp1 (lysosomal marker). (b) Lysosomal staining of the NAGLU-silenced stable clone (ΔNAGLU): ΔNAGLU clone seeded on coverslips was grown for 48 h in the presence of 20 μM of each l-iminosugar and then processed as for the CTRL clone. Magnifications are relative to the dashed white squares. (c,d) Quantification of immunofluorescence staining: the histograms represent, respectively, the quantification of cells with enlarged Lamp1 positive structures (bottom left) and the mean fluorescence intensity of Lamp1 (bottom right) among the different experimental conditions. 50 randomly chosen cells from three independent experiments were used for quantification. Single focal sections are shown. Scale bar: 20 μm. Asterisks indicate the statistically significant differences: (**) *p*-value < 0.001, (***) *p*-value < 0.0001.

In order to investigate the effect of iminosugars *ent*-(**1**–**7**) in cellular models of Sanfilippo disease, we used first a stable clone of the SK-NBE human neuroblastoma cell line silenced for *NAGLU* gene (ΔNAGLU), as a neuronal cell model of Sanfilippo B disease, which we recently generated in our lab.^45^ This clone fully recapitulates the lysosomal phenotype of Sanfilippo B affected cells, thus providing a useful tool for *in vitro* testing the therapeutic efficacy of novel drug candidates. Indeed, silencing of *NAGLU* gene caused accumulation of enlarged lysosomes (Lamp1-positive structures) into the cytoplasm of SK-NBE ΔNAGLU clone as compared to control clone (CTRL) stable transfected with a nontargeting shRNA (Figure 2). SK-NBE ΔNAGLU and CTRL clones were selected to test the effect of iminosugars on the lysosomal phenotype. Both clones were grown in the presence of 20 μM of each l-iminosugar *ent*-(**1**–**7**) in normal growth conditions, and, after 48 h, the lysosomal accumulation was evaluated by Lamp1 staining and compared to untreated clones (Figure 2). Treatment with any of the seven l-iminosugars did not cause any change in lysosomal size and distribution of nondiseased clone (CTRL) (Figure 2a). Further assays (apoptosis and anti-proliferative effect) performed by us confirmed that *ent*-(**1**–**7**) did not induce any cytotoxic effect in different cell lines (IB3-1, HaCaT, and CuFi) at concentrations up to 100 μM (unpublished data). Moreover, while untreated ΔNAGLU clone showed accumulation of enlarged Lamp1 positive lysosomal structures within the cytoplasm, after treatment with l-iminosugars *ent*-(**1**–**7**), a significant reduction of lysosomal enlargement in ΔNAGLU clone was observed in the presence of compounds *ent*-**1**, *ent*-**2**, *ent*-**6**, and *ent*-**7** (Figure 2b).

No effect, instead, was highlighted with *ent*-(**3**–**5**). Moreover, the distribution of lysosomes inside the cytoplasm of ΔNAGLU clone treated with *ent*-**1**, *ent*-**2**, *ent*-**6** and *ent*-**7** appeared to be no more concentrated in the perinuclear region as they are in the untreated NAGLU-silenced clone. Indeed, as previously described by us and others, lysosomes are trapped in the perinuclear region in different lysosomal disease models.^49^ In our Sanfilippo B model cell, lysosomes appeared physiologically dispersed all over the cytoplasm upon treatment with the active l-iminosugars *ent*-**1**, *ent*-**2**, *ent*-**6**, and *ent*-**7**. Physiological distribution of the lysosomes in the ΔNAGLU clone treated with *ent*-**1**, *ent*-**2**, *ent*-**6**, and *ent*-**7** is more evident if we compare Lamp1 immunostaining of these cells with control normal clone (CTRL), where lysosomes occupy all the available cytoplasm and periphery of the cells.

**Figure 3:**
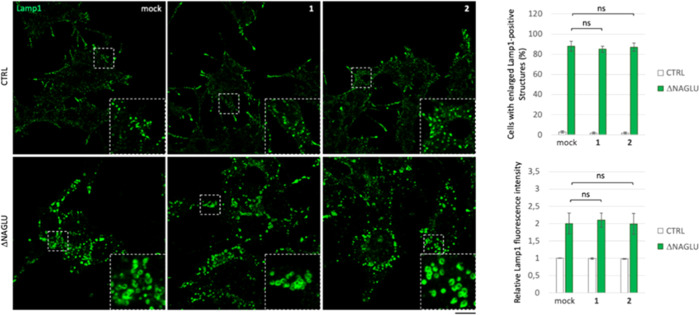
Effect of d-iminosugars in rescuing the lysosomal phenotype in the Sanfilippo B cellular model. Lysosomal staining of control clone and NAGLU silenced stable clone (ΔNAGLU) treated with iminosugars **1** and **2**: stable clones seeded on coverslips were grown for 48 h in the presence of 20 μM of each d-iminosugar and then processed for indirect immunofluorescence by using a specific antibody against Lamp1 (lysosomal markers). Quantification of immunofluorescence staining: histograms represent, respectively, the quantification of cells with an enlarged Lamp1 positive structure (bottom left) and the mean fluorescence intensity of Lamp1 (bottom right) among the different experimental conditions. 50 randomly chosen cells from three independent experiments were used for quantifications. Single focal sections are shown. Scale bar: 20 μm. (ns) *p*-value not statistically significant.

To evaluate whether chirality of the iminosugar moiety exerted a key role in the observed activity, the well-known d-DNJ (**1**) and NBDNJ (**2**) were then considered. To this purpose, both CTRL and ΔNAGLU clones were grown in the presence of 20 μM of each d-iminosugar in normal growth conditions, and after 48 h the lysosomal phenotype was evaluated by Lamp1 staining as compared to untreated clones (Figure 3). Treatment with both **1** and **2** did not cause any changes in the lysosomal size and distribution in both nondiseased model cells (CTRL) and Sanfilippo B disease model ΔNAGLU clone (Figure 3), thereby demonstrating that the efficacy highlighted by our compounds was closely related to the configuration of the pseudo-sugar moiety.

We recently demonstrated the pathological accumulation of HS on the cell membrane of SK-NBE ΔNAGLU clone and Sanfilippo B patient-derived fibroblasts as well.^45^ Thus, to assess whether l-iminosugars would also be able to affect HS accumulation on the cell surface of ΔNAGLU clone, we performed indirect immunofluorescence using the anti-HS 10E4 antibody that recognizes the extracellular accumulated HS.

**Figure 4:**
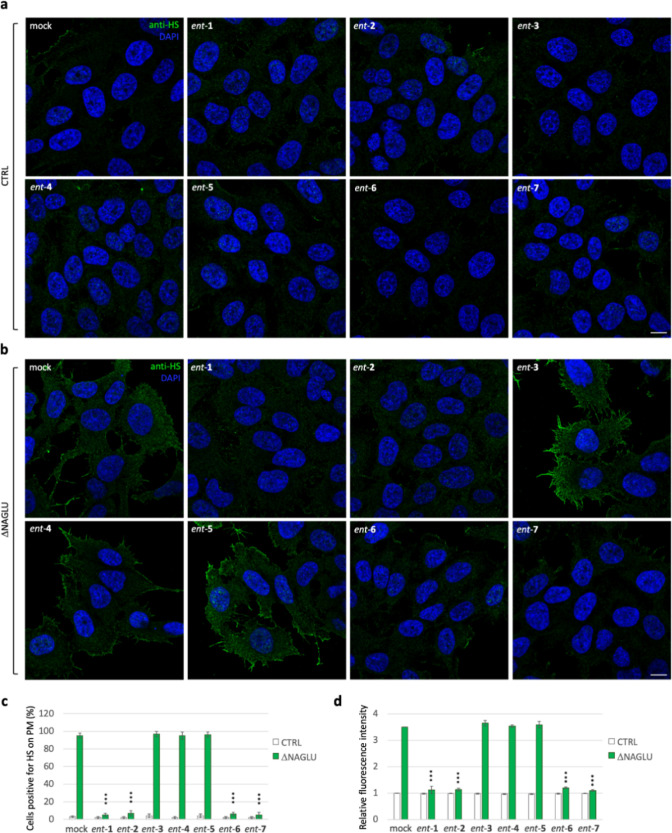
Activity of *ent*-(**1**–**7**) in rescuing the HS accumulation in the ΔNAGLU clone. (a) HS staining of CTRL clone treated with *ent*-(**1**–**7**): control stable clone seeded on coverslips was grown for 48 h in the presence of 20 μM of each l-iminosugar and then processed for indirect immunofluorescence by using the anti-HS antibody 10E4. (b) HS staining of ΔNAGLU clone treated with *ent*-(**1**–**7**): ΔNAGLU clone seeded on coverslips was grown for 48 h in the presence of 20 μM of each l-iminosugar and then processed as for the CTRL clone. (c,d) Quantification of immunofluorescence staining: histograms represent, respectively, (c) the quantification of cells positive for HS on plasma membrane (%) and (d) the mean fluorescence intensity of HS among the different experimental conditions. 50 randomly chosen cells from three independent experiments were used for quantification. Single focal sections are shown. Scale bar: 10 μm. Asterisks indicate the statistically significant differences: (***) *p*-value < 0.0001.

To this end, CTRL and ΔNAGLU clones were grown in the presence of 20 μM of each l-iminosugar *ent*-(**1**–**7**) in normal growth conditions, and after 48 h HS accumulation was evaluated by immunofluorescence staining (Figure 4). While CTRL clone did not show any HS accumulation (Figure 4a), the untreated ΔNAGLU clone showed consistent HS staining on cell membrane (Figure 4b). A prominent reduction of HS staining in ΔNAGLU clone was instead observed in the presence of *ent*-**1**, *ent*-**2**, *ent*-**6**, and *ent*-**7** (Figure 4b). Noteworthy, also in this case, *ent*-**3**, *ent*-**4**, and *ent*-**5** were ineffective in reducing HS accumulation in ΔNAGLU clone, in line with the results already obtained with the lysosomal staining. Furthermore, treatment with any of the seven l-iminosugars did not cause any change in the HS staining of the nondiseased cells (CTRL) (Figure 4a).

Overall, these results show, for the first time, that treatment with *ent*-**1**, *ent*-**2**, *ent*-**6**, and *ent*-**7** is able to rescue both lysosomal phenotype and pathological HS accumulation on cell membrane of a cellular model of Sanfilippo B disease.

#### l-Iminosugars Reduce HS Accumulation and Correct Lysosomal Defects in Sanfilippo B Patient Fibroblasts

**Figure 5:**
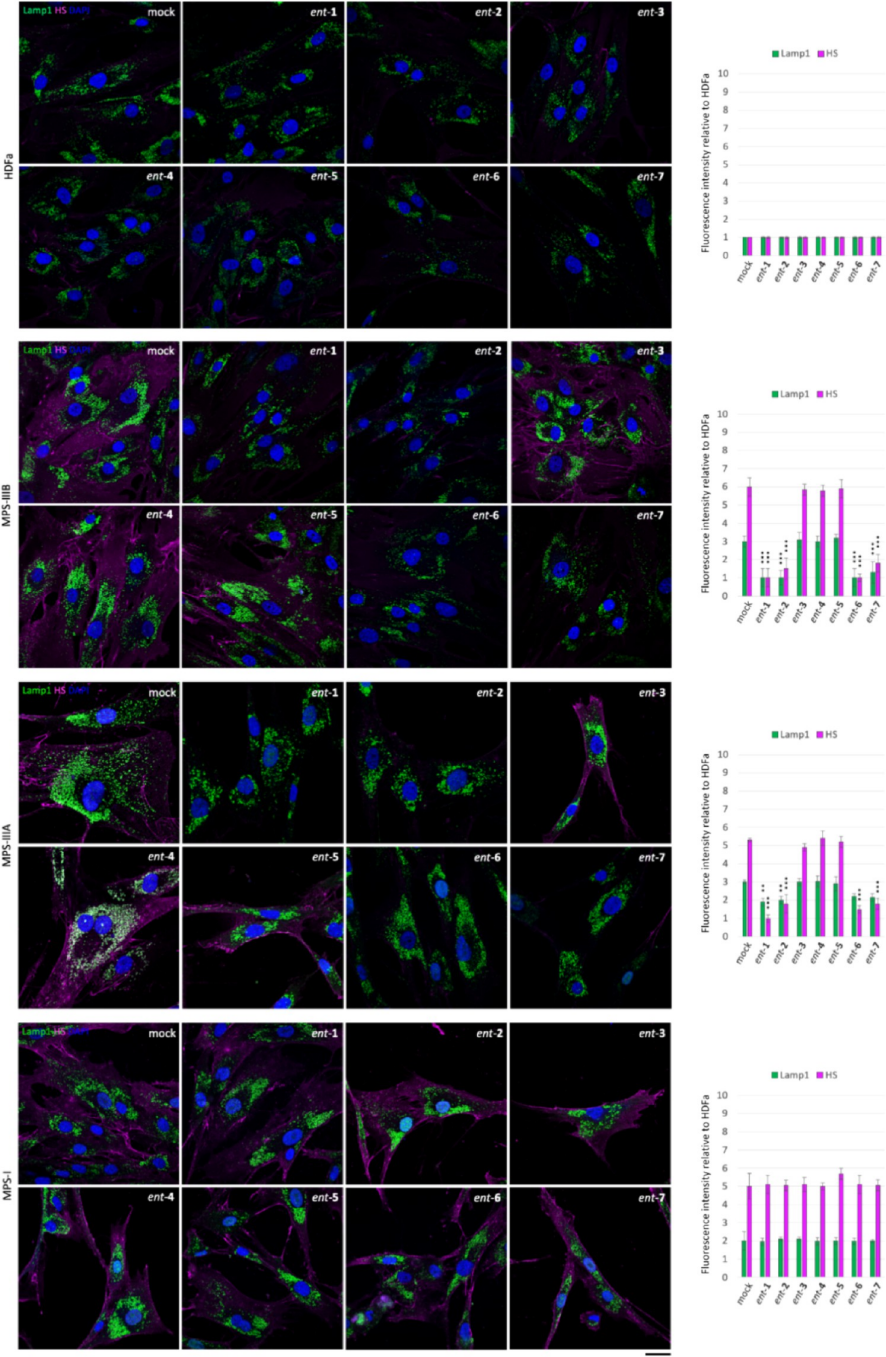
Activity of iminosugars *ent*-(**1**–**7**) in rescuing lysosomal phenotype and HS accumulation in Sanfilippo B and A fibroblasts. HS and Lamp1 staining of the HDFa and Sanfilippo B (MPS IIIB), Sanfilippo A (MPS IIIA), and MPS I fibroblasts treated with *ent*-(**1**–**7**): fibroblasts seeded on coverslips were grown for 48 h in the presence of 20 μM of each l-iminosugar and then processed for indirect immunofluorescence by using specific antibodies against HS and Lamp1 and decorated with DAPI (nuclear marker). Quantifications of immunofluorescence staining: the histograms represent the quantification of the mean fluorescence intensity of each MPS sample relative to the untreated cells. 50 randomly chosen cells from three independent experiments were used for quantifications. Single focal sections are shown. Scale bar: 50 μm. Asterisks indicate the statistically significant differences: (***) *p*-value < 0.0001.

In order to test whether l-iminosugars would exert the same effects also on human patient cells, we selected Sanfilippo B-derived fibroblasts as disease model^50^ and human adult dermal fibroblasts HDFa as control. Fibroblasts were grown on a coverslip in the presence of *ent*-(**1**–**7**) at the dosage of 20 μM, and, after 48 h, processed for both HS and Lamp1 immunostaining (Figure 5). As shown, treatment with l-iminosugars did not have any effect on HDFa control fibroblasts. On the other hand, *ent*-**1**, *ent*-**2**, *ent*-**6**, and *ent*-**7** caused a strong reduction of HS accumulated on the cell membrane of Sanfilippo B (MPS IIIB) fibroblasts (Figure 5).

Notably, the same compounds exerted a strong effect also on the lysosomal phenotype as highlighted above in ΔNAGLU clone. No effect, instead, was observed in the presence of iminosugars *ent*-**3**, *ent*-**4**, and *ent*-**5** (Figure 5).

**Figure 6:**
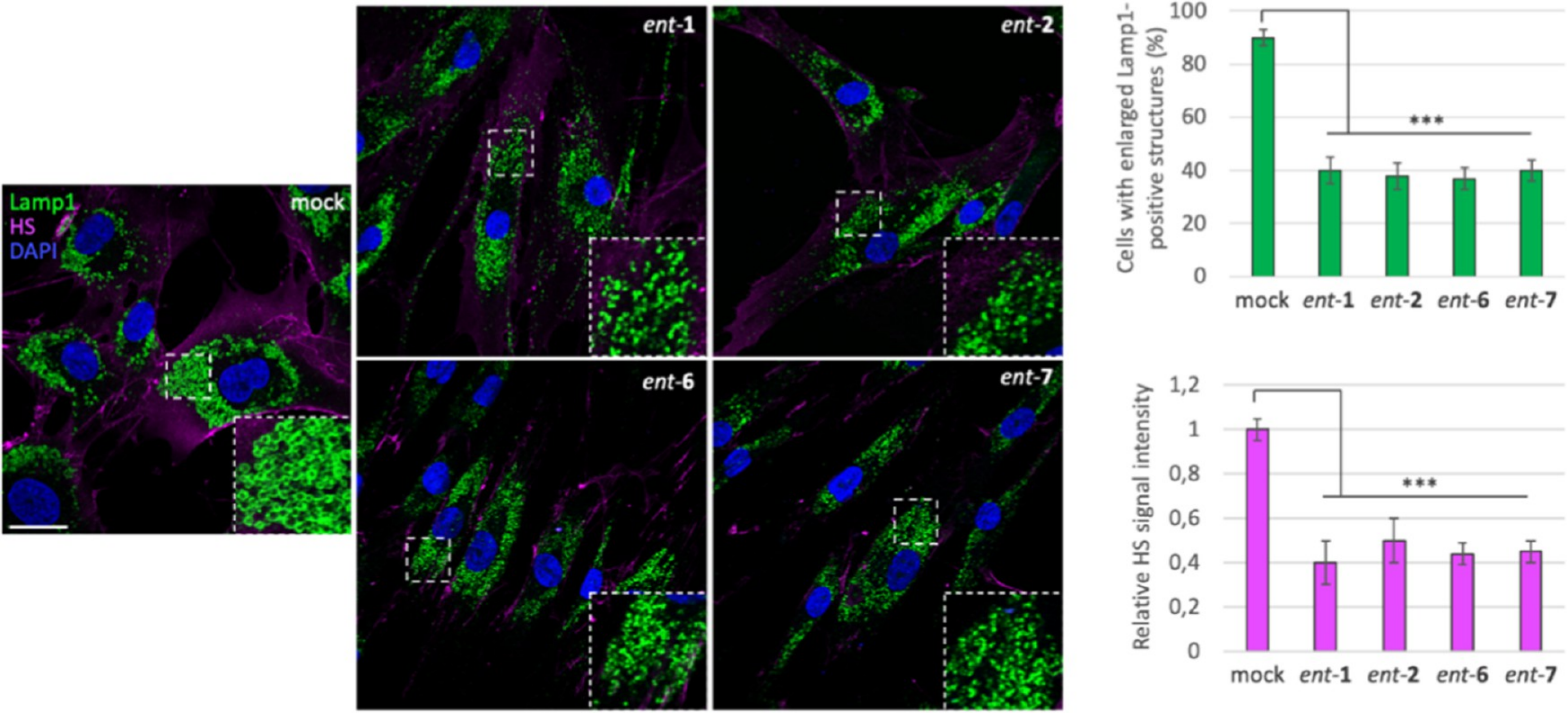
Activity of *ent*-**1**, *ent*-**2**, *ent*-**6**, and *ent*-**7** in rescuing the lysosomal phenotype and HS accumulation in fibroblasts from MPS I patients. HS and Lamp1 staining of the l-iminosugar-treated and -untreated MPS I fibroblasts. Fibroblasts seeded on coverslips were grown for 48 h in the presence of 40 μM of each l-iminosugar, then processed for indirect immunofluorescence by using specific antibodies against HS and Lamp1, and decorated with DAPI (nuclear marker). Quantification of immunofluorescence staining: histograms represent the quantification of the mean fluorescence intensity of each MPS sample relative to untreated cells. 50 randomly chosen cells from three independent experiments were used for the quantification. Single focal sections are shown. Scale bar: 50 μm. Asterisks indicate the statistically significant differences: (***) *p*-value < 0.0001.

Since we found a strong reduction of HS staining in Sanfilippo B fibroblasts, we investigated whether treatment with the same compounds would have an effect on other lysosomal diseases with HS accumulation like Sanfilippo A (MPS IIIA) and MPS I (Figure 5). We treated fibroblasts obtained from Sanfilippo A (MPS IIIA)- and MPS I-affected patients^50^ with the same compounds and dosage. Interestingly, a reduction of HS staining and lysosomal enlargement was observed by treatment with only *ent*-**1**, *ent*-**2**, *ent*-**6**, and *ent*-**7** also in Sanfilippo A-patient-derived fibroblasts (Figure 5). Conversely, l-iminosugars were unable to reduce HS and rescue lysosomal defects of MPS I fibroblasts, where the distribution and fluorescence intensity of lysosomes did not change as compared to untreated MPS I fibroblasts (Figure 5). Notably, a reduction of HS staining and lysosomal defects was detectable in MPS I fibroblasts only if the concentration of *ent*-**1**, *ent*-**2**, *ent*-**6**, and *ent*-**7** was doubled to 40 μM (Figure 6).

These results suggest that treatment with iminosugars *ent*-**1**, *ent*-**2**, *ent*-**6**, and *ent*-**7** is able to rescue HS and lysosome accumulation in Sanfilippo A- and B-patient-derived fibroblasts, while a higher concentration of l-iminosugars is required to affect MPS I diseased fibroblasts probably due to the accumulation of an additional GAG, the dermatan sulfate (DS), besides HS, and to the severity of the disease.

**Figure 7:**
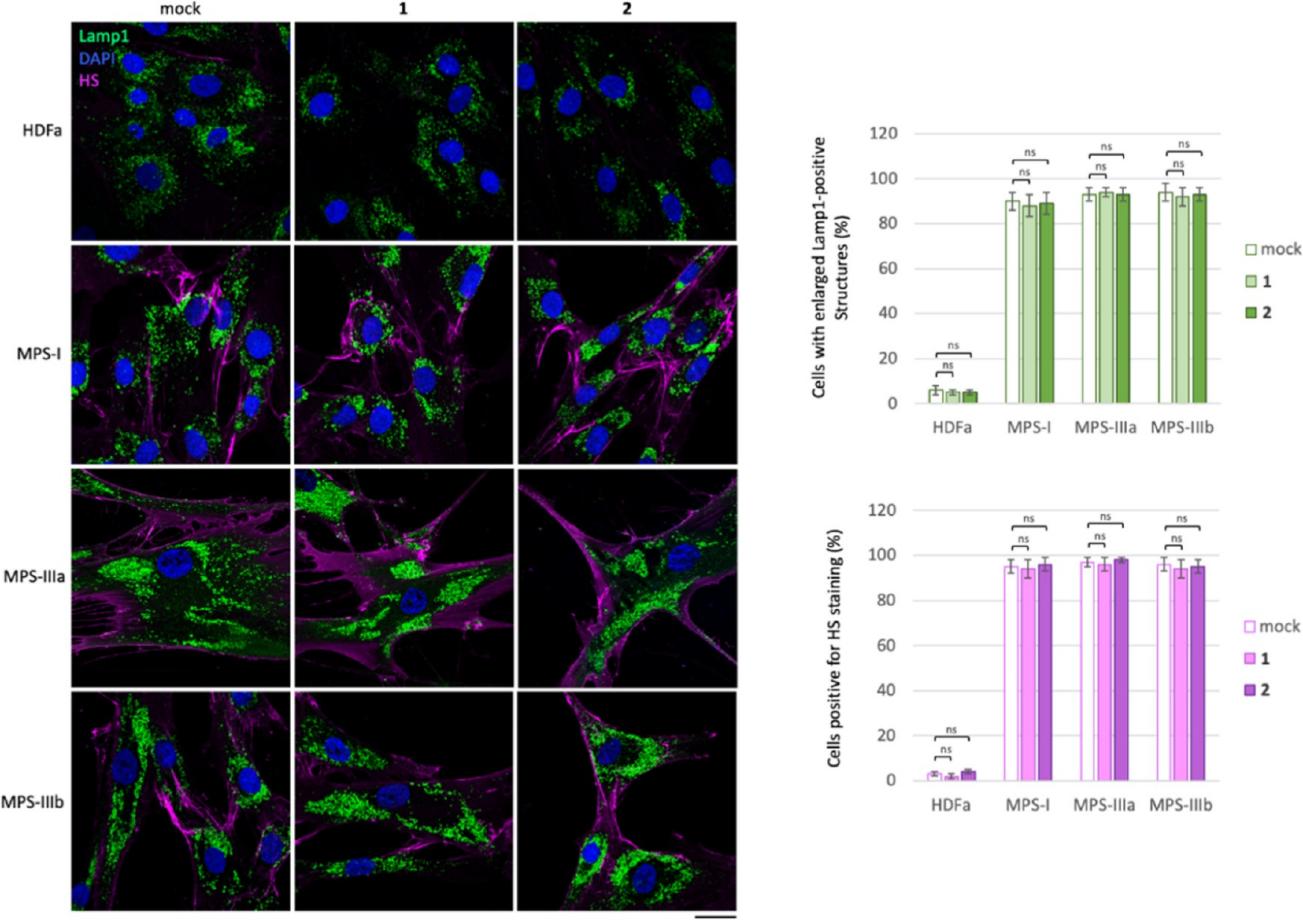
No effect of d-iminosugars in rescuing the lysosomal phenotype in fibroblasts from MPS patients. HS and Lamp1 staining of d-iminosugars-treated and -untreated HDFa, MPS I, MPS IIIA, and MPS IIIB fibroblasts. Fibroblasts seeded on coverslips were grown for 48 h in the presence of 20 μM of each d-iminosugar, then processed for indirect immunofluorescence by using specific antibodies against HS and Lamp1, and decorated with DAPI (nuclear marker). Quantification of immunofluorescence staining: histograms represent the quantification based on the percentage of cells positive for enlarged Lamp1 structures and HS staining. 50 randomly chosen cells from three independent experiments were used for quantification. Single focal sections are shown. Scale bar: 50 μm. Asterisks indicate the statistically significant differences: (ns) *p*-value not statistically significant.

Finally, to further assess the role of iminosugar chirality, we tested the efficacy of d-enantiomers **1** and **2** in reducing lysosomal defects and HS accumulation also in patient fibroblasts. Sanfilippo A- and B-patient-derived fibroblasts, MPS I, and human adult dermal (HDFa) fibroblasts were grown on a coverslip in the presence of iminosugars **1** and **2** at the dosage of 20 μM and, after 48 h, processed for both HS and Lamp1 immunostaining. As shown in Figure 7, treatment with **1** and **2** did not have any effect on either lysosomal size and distribution or extracellular HS accumulation in both control and diseased fibroblasts.

Overall, these results indicate that, differently from **1** and **2**, iminosugars *ent*-**1**, *ent*-**2**, *ent*-**6**, and *ent*-**7**, represent promising compounds worthy of further investigation in the fight against Sanfilippo B subtype and also for Sanfilippo A and MPS I.

#### l-Iminosugars Cause Reduction of HS Levels in Highly HS-Decorated HeLa Cells

In order to test whether treatment with *ent*-(**1**–**7**) would exert the same effects on HS levels in a cell line where *NAGLU* gene is not mutated, we selected the HeLa human epithelial cancer cell line that is highly decorated by HS on the cell surface. Cells were grown for 48 h in the presence of *ent*-(**1**–**7**), and the quantity of HS was evaluated by immunostaining with the specific anti-HS antibody 10E4.^45^ In accordance with all the above described results, only treatment with *ent*-**1**, *ent*-**2**, *ent*-**6**, and *ent*-**7** caused a reduction of the cell membrane HS (Figure S1). l-Iminosugars *ent*-**3**, *ent*-**4**, and *ent*-**5** were also inactive in HeLa cells as in the other cellular tools used in previous experiments. Moreover, since HS is fundamental to mediate growth factor activity, we asked whether HS reduction on the cell surface would interfere with cell growth of HeLa cancer cell line. Consistent with our hypothesis, the number of HeLa cells decreased when cells were grown under treatment for 48 h with 20 μM of the active iminosugars *ent*-**1**, *ent*-**2**, *ent*-**6**, and *ent*-**7** as compared to the untreated HeLa cells, while *ent*-**3**, *ent*-**4**, and *ent*-**5**, together with **1** and **2**, did not show any significant effect on HeLa cell number count (Figure S2a). The results herein obtained suggest that, as HeLa cells hold a nonmutated NAGLU enzyme, the HS reduction observed could be ascribed to the ability of the active iminosugars to downregulate HS synthesis.^51^ Therefore, we next investigated whether treatment with the active l-iminosugars would exert their effects on HS through the reduction of protein levels of two key enzymes involved in HS synthesis, namely exostosin glycosyltransferase, EXT1, and EXT2,^52^ or of the core protein of the HS proteoglycan syndecan2, SDC2.^52^ We performed Western blotting analyses to evaluate protein levels of EXT1, EXT2, and SDC2 in HeLa cells untreated and treated with 20 μM of *ent*-**1**, *ent*-**2**, *ent*-**6**, and *ent*-**7** for 24 h (Figure S2b). The results obtained show that only *ent*-**1** and *ent*-**2** caused a significant decrease in protein levels of both EXT1 and EXT2 enzymes. Remarkably, treatment with *ent*-**6** and *ent*-**7** had a different effect on protein levels of these two enzymes, suggesting that their mechanism of action might be different from that of *ent*-**1** and *ent*-**2**. Moreover, none of these four l-iminosugars had a relevant effect on the levels of SDC2 proteoglycan core protein.

These results suggest that the effects of *ent*-**1** and *ent*-**2** in reducing HS could be attributable to their ability to reduce protein levels of HS synthetic enzymes EXT1 and EXT2 in cells with a full active NAGLU enzyme.

#### l-Iminosugars Increase NAGLU Protein Levels and Enzymatic Activity

**Figure 8:**
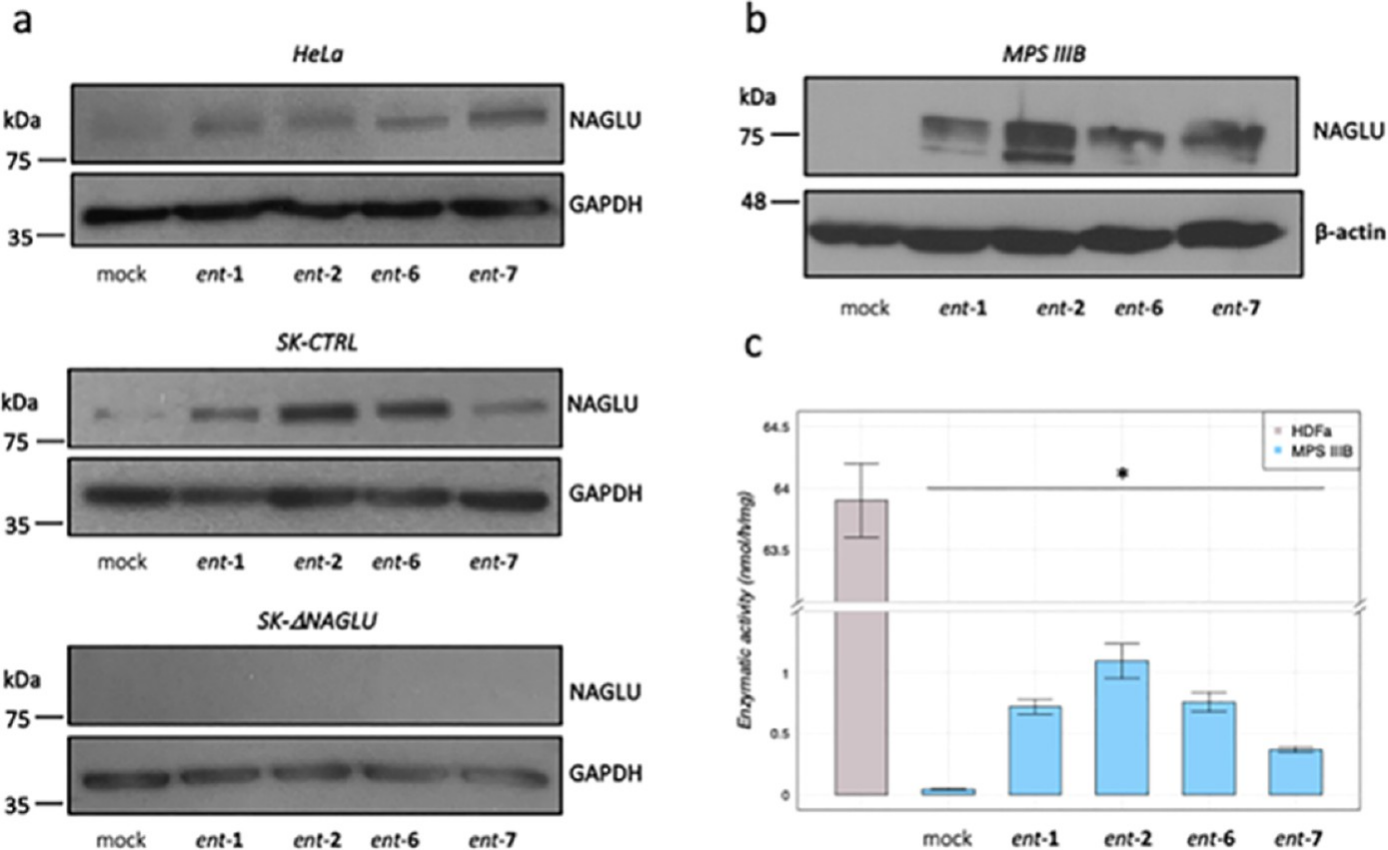
Effect of l-iminosugars on NAGLU protein levels and enzymatic activity. (a) Western blotting analysis of NAGLU protein levels in untreated mock (lane 1) and treated with *ent*-**1** (lane 2), *ent*-**2** (lane 3), *ent*-**6** (lane 4), and *ent*-**7** (lane 5) HeLa cells, SK-NBE clones (CTRL), and NAGLU silenced clone. To monitor equal loading of the proteins in the gel lanes, the blots were re-probed using an anti-GAPDH antibody. (b) Western blotting analysis of NAGLU protein levels in untreated and treated fibroblasts from MPS IIIB patients. To monitor equal loading of the proteins in the gel lanes, the blots were re-probed using an anti-β-actin antibody. (c) Enzymatic activity of mutated NAGLU extracted from Sanfilippo B fibroblasts: extracts from HDFa (control) and Sanfilippo B fibroblasts were incubated or not with 20 μM of l-iminosugars together with 4-methylumbelliferyl-*N*-acetyl-α-d-glucosaminide as the fluorogenic substrate.

In order to eventually provide mechanistic insights into the activity of our l-iminosugars, we evaluated first whether the reduction of HS in the diseased Sanfilippo B clone and HeLa cells would be due to the up-regulation of HS lysosomal degradative enzyme, NAGLU. Therefore, we investigated the levels of NAGLU protein in HeLa cells by Western blotting analysis and in both nondiseased model cells (CTRL) and NAGLU silenced SK-NBE (ΔNAGLU) clone. As shown in Figure 8a (upper panel) treatment of HeLa cells with 20 μM of *ent*-**1**, *ent*-**2**, *ent*-**6**, and *ent*-**7** for 24 h caused a significant increase in NAGLU protein levels. Remarkably, a similar effect on NAGLU protein levels was observed in the SK-NBE CTRL clone upon treatment with the same l-iminosugars (Figure 8a, middle panel). These results suggest that the activity of these l-iminosugars in reducing HS levels in the tested cell models could be attributable to the increase in NAGLU protein levels. However, although this mechanism appears to occur in HeLa cells, this is only in part true for our Sanfilippo B model system. Treatment of SK-NBE NAGLU silenced (ΔNAGLU) clone with 20 μM of *ent*-**1**, *ent*-**2**, *ent*-**6**, and *ent*-**7** for 24 h did not cause any increase in NAGLU protein levels (Figure 8a, lower panel), since the expression of the NAGLU interfering RNA in this clone completely abolishes NAGLU expression. Therefore, these results indicate that the mechanism of action of the selected l-iminosugars might not be related only to the increase of NAGLU protein levels. In order to evaluate the effect of the active l-iminosugars on NAGLU protein levels also in fibroblasts from Sanfilippo B patients, we treated those cells (MPS IIIB) for 24 h with 20 μM of *ent*-**1**, *ent*-**2**, *ent*-**6**, and *ent*-**7**. Remarkably, while untreated Sanfilippo B fibroblasts did not show any band for NAGLU protein, since mutated unfolded proteins are degraded by the proteasome, upon treatment with the active l-iminosugars, an increased level of NAGLU protein was detectable (Figure 8b).

*In vitro* enzymatic assay for the mutated NAGLU extracted from Sanfilippo B fibroblasts was then performed to establish if the iminosugars *ent*-**1**, *ent*-**2**, *ent*-**6**, and *ent*-**7** would be able to increase NAGLU activity. To this aim, HDFa (CTRL) and Sanfilippo B extracts were used in the absence or presence of iminosugars *ent*-**1**, *ent*-**2**, *ent*-**6**, and *ent*-**7** (20 μM) and of 4-methylumbelliferyl-*N*-acetyl-α-d-glucosaminide as fluorogenic substrate^53,54^ to measure NAGLU enzymatic activity. Normalizing for total protein concentration, HDFa showed normal NAGLU enzymatic activity, while Sanfilippo B fibroblasts had almost zero enzymatic activity as a result of the absence of NAGLU protein. The addition of *ent*-**1**, *ent*-**2**, *ent*-**6**, and *ent*-**7** to the reaction mixture induced an up to 20 fold increase of NAGLU enzymatic activity in the case of *ent*-**2** (Figure 8c), suggesting therefore that these active l-iminosugars might act in stabilizing the mutated enzyme.

Moreover, in order to assess if the increase of NAGLU enzymatic activity could be ascribed to the interaction of iminosugars with the enzyme active site, their inhibitory effect toward nonmutated NAGLU activity was evaluated. As a result, when *ent*-**1**, *ent*-**2**, *ent*-**6,** and *ent*-**7** were added to the reaction mix of the HDFa lysate up to a concentration of 1 mM, no inhibition was observed, suggesting that iminosugars-NAGLU interaction involved a site different from the active one.

#### l-Iminosugars Rescue Proper Sorting of NAGLU Protein toward Lysosomal Compartment

**Figure 9:**
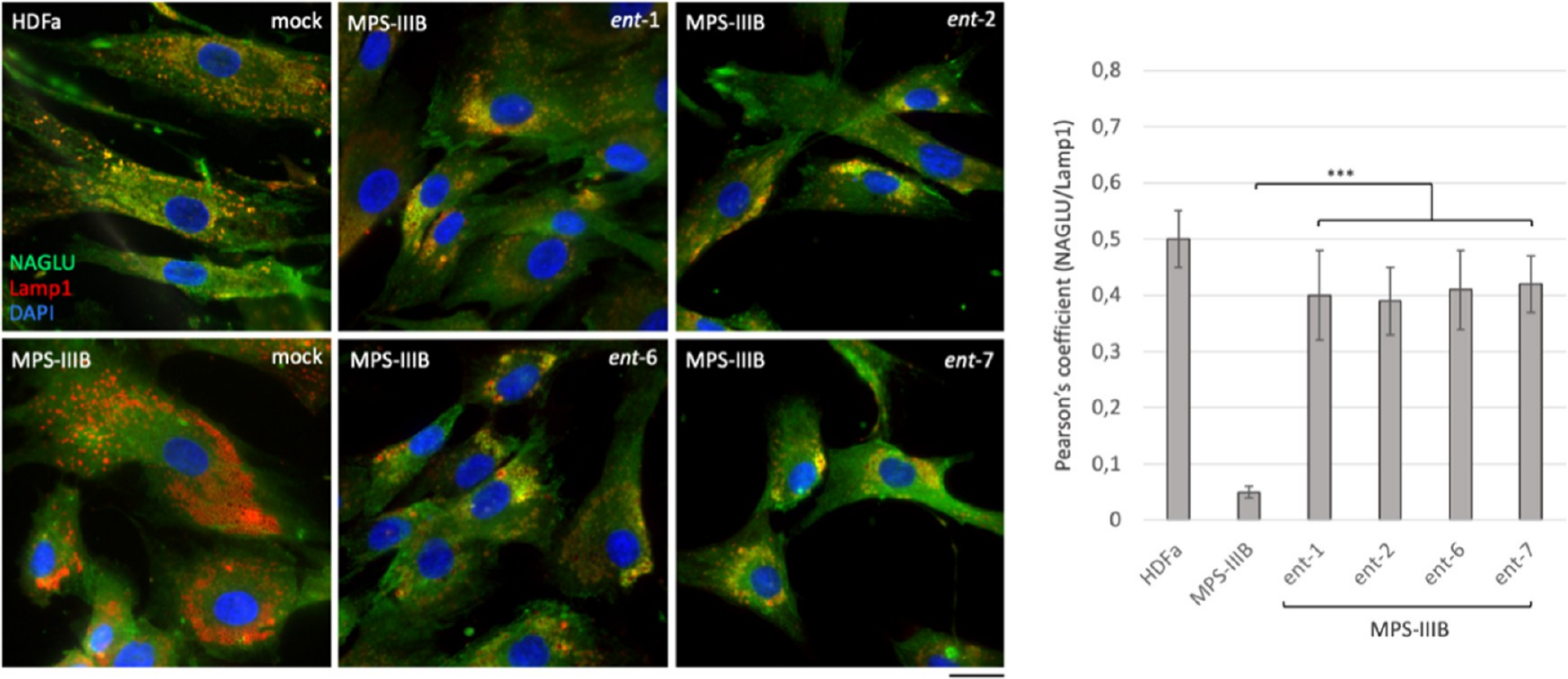
Effect of the active l-iminosugars on NAGLU sorting to the lysosomal compartment. HDFa and MPS IIIB seeded on coverslips were grown for 48 h in the presence or not (mock) of 20 μM of *ent*-**1**, *ent*-**2**, *ent*-**6,** and *ent*-**7**, then processed for indirect immunofluorescence by using specific antibodies against NAGLU and Lamp1, and decorated with DAPI (nuclear marker). Quantification of immunofluorescence staining: histograms represent the quantification of the mean fluorescence intensity of each MPS IIIB sample relative to the untreated cells. 50 randomly chosen cells from three independent experiments were used for quantifications. Single focal sections are shown. Scale bar: 50 μm. Asterisks indicate the statistically significant differences: (***) *p*-value < 0.0001.

We next looked at the effect of the active l-iminosugars on lysosomal sorting of NAGLU enzyme. Fibroblasts (HDFa and MPS IIIB) were incubated in the presence of 20 μM of each active l-iminosugar in normal growth conditions and, after 48 h, co-localization of NAGLU with the lysosomal associated membrane protein 1 (Lamp1) was analyzed by confocal microscopy. HDFa showed normal distribution of NAGLU enzyme within the cytoplasm co-localizing with the lysosomes, as indicated by the yellow dots. On the other hand, MPS IIIB fibroblasts showed a substantial pathological accumulation of enlarged Lamp1 positive organelles and the absence of NAGLU co-staining. Strikingly, when MPS IIIB fibroblasts were treated with *ent*-**1**, *ent*-**2**, *ent*-**6,** and *ent*-**7**, NAGLU enzyme was found to co-localize with lysosomes (Figure 9). This result was confirmed by quantitative analysis of the total NAGLU/Lamp1 positive structures. Overall, these data further support the ability of the active iminosugars to properly sort the mutated NAGLU enzyme toward the lysosomal compartment.

#### l-Iminosugars Reduce Amyloid Peptide Aβ1-42 Accumulation in the Cytoplasm of ΔNAGLU Clone

**Figure 10:**
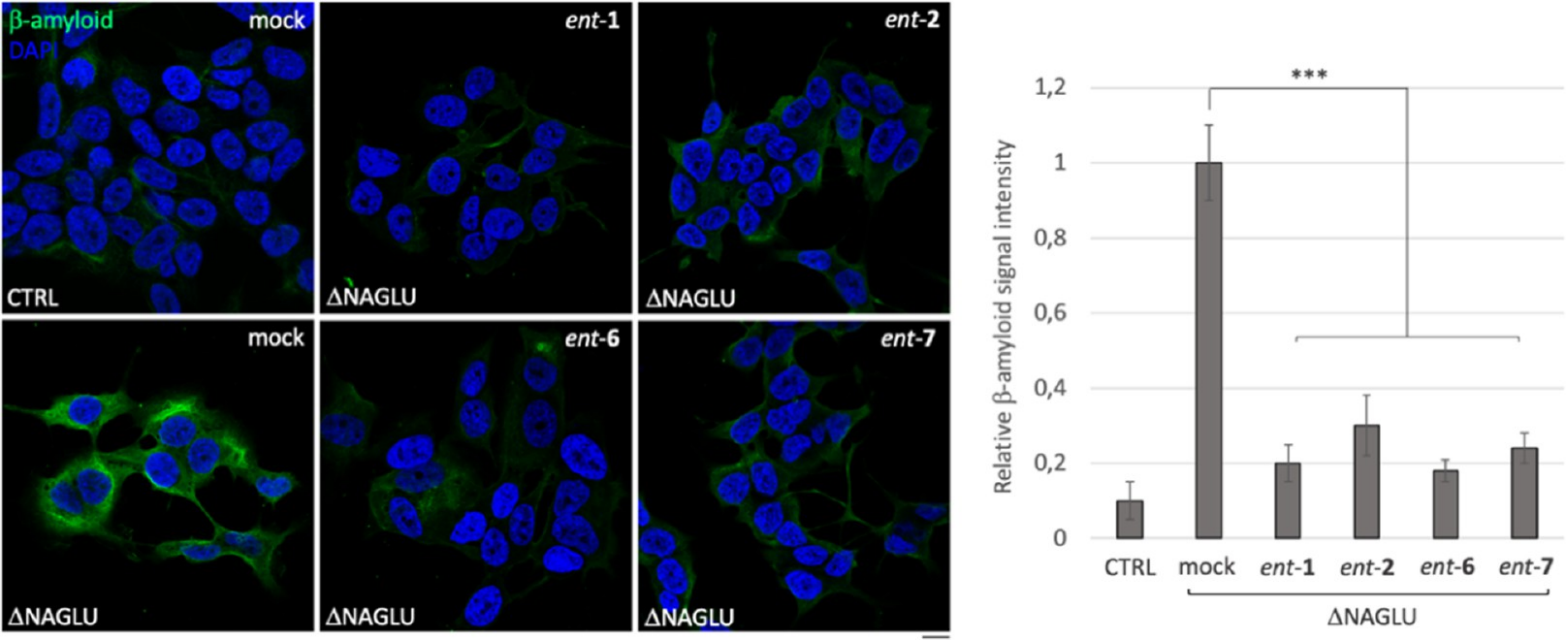
Activity of *ent*-**1**, *ent*-**2**, *ent*-**6**, and *ent*-**7** in rescuing the accumulation of Aβ1-42 peptide in the Sanfilippo B cellular model. Aβ1-42 peptide immunostaining of control (CTRL) and ΔNAGLU clone untreated and treated with *ent*-**1**, *ent*-**2**, *ent*-**6**, and *ent*-**7**: ΔNAGLU clone seeded on the coverslips were grown for 48 h in the presence of 20 μM of each l-iminosugar and then processed for indirect immunofluorescence by using a specific Aβ1-42 peptide antibody. Quantification of immunofluorescence staining: the histograms represent, respectively, the quantification of cells with Aβ1-42 peptide positive structures among the different experimental conditions. 50 randomly chosen cells from three independent experiments were used for quantification. Single focal sections are shown. Scale bar: 10 μm. Asterisks indicate the statistically significant differences: (***) *p*-value < 0.0001.

Multiple amyloid proteins including α-synuclein, prion protein (PrP), Tau, and amyloid β progressively aggregate in the brain of Sanfilippo patients and murine models,^55,56^ resulting in a key pathogenic mechanism that contributes to the development of the neurological phenotype in Sanfilippo B patients. Therefore, we asked whether the administration of *ent*-**1**, *ent*-**2**, *ent*-**6**, and *ent*-**7** would influence the deposition of Aβ1-42 peptide in the cytoplasm of ΔNAGLU clone. To this aim, ΔNAGLU clone was grown in the presence of 20 μM of each active l-iminosugar in normal growth conditions, and after 48 h the accumulation of Aβ1-42 peptide was evaluated by immunostaining and compared to that of the untreated clone (Figure 10). Interestingly, for the first time, we demonstrate that in an *in vitro* model of Sanfilippo disease, the ΔNAGLU clone accumulates the amyloid peptide Aβ1-42 within the cytoplasm.

Moreover, treatment with the four active l-iminosugars caused a drastic reduction of the Aβ1-42 peptide accumulation in ΔNAGLU clone as compared to the untreated one (Figure 10).

Overall, these results suggest that treatment with *ent*-**1**, *ent*-**2**, *ent*-**6**, and *ent*-**7** could be an effective tool not only for preventing the primary cause of the disease, such as HS accumulation and lysosomal defects, but also to regulate other pathogenic mechanisms that contribute to the progression of the neurological phenotype of Sanfilippo syndrome.

## Discussion and Conclusions

Despite clinical improvements achieved through ERT, SRT, or other therapies in patients affected by various types of LSDs, the need for new therapeutic approaches for Sanfilippo syndrome, which presents a severe neurological involvement, persists. Recently, iminosugars emerged as attractive therapeutic agents for LSDs due to their capability to penetrate the blood–brain barrier, be orally bioavailable, and have a broad tissue distribution. In the frame of our studies on the identification of the relationship between the chirality of iminosugars and their therapeutic potential, we described the activity of *N*-butyl-l-deoxynojirimycin (*ent*-**1**) and some other *N*-alkylated l-iminosugars in Pompe disease and cystic fibrosis lung disease. Herein, we demonstrated the efficacy of four synthetic iminosugars, belonging to the unnatural l-series,^40^ in reducing substrate accumulation and lysosomal defects in a neuronal cell model of Sanfilippo B disease and in fibroblasts derived from Sanfilippo B-, Sanfilippo A-, and MPS I-affected patients.

Reduction of accumulated substrate in the lysosomes and/or other cellular compartments is the goal of all the developed therapies for LSDs. Indeed, substrate accumulation triggers a redistribution of the lysosomes, their expansion, and remarkable defects in their functions, including degradative activity, trafficking, and secretion.^49,57−59^ In particular, an impaired lysosomal secretion, which represents a hallmark common to most LSDs, has been associated with the progressive accumulation of secondary substrates occurring in these pathologies.^26,60^ Indeed, high levels of gangliosides GM2 and GM3 have been found in the brain of Sanfilippo patients, as well as in other MPS types and a variety of other LSDs.^61^ Accumulation of secondary metabolites contributes to the pathophysiology of LSDs through multiple mechanisms which include impairment of vesicle trafficking and fusion of cellular membranes.^62^ In addition, it is worth pointing out that in Sanfilippo syndrome and other MPS types, primary substrate accumulation is not only restricted to the lysosomes but also redistributed to various cellular and extracellular compartments.^53,63−69^

Multiple evidence also highlight the role of HS localized at the cell surface or extracellularly in the progression of neurological manifestations in various MPS types, including Sanfilippo diseases,^63,70^ thus paving the way to novel therapeutic approaches for these diseases.^71^

In this work, we demonstrated the capability of four iminosugars, *ent*-**1**, *ent*-**2**, *ent*-**6**, and *ent*-**7**, in reducing HS accumulation in cellular models of Sanfilippo disease. Reduction of substrate accumulation in cells triggered by the active iminosugars resulted in an attenuation of the lysosomal pathologic phenotype. The activity of these compounds is strictly dependent on iminosugar chirality, as demonstrated by the observation that the iminosugars duvoglustat (**1**) and miglustat (**2**), the enantiomers of active *ent*-**1** and *ent*-**2**, did not show any effect on HS storage and lysosomal defects in our cellular models. Thus, encouraged by these results, we performed preliminary mechanistic studies of the active iminosugars capable of restoring a normal phenotype in MPS IIIB diseased cells. In particular, from Western blotting analysis, the l-enantiomers of **1** and **2** (*ent*-**1** and *ent*-**2**) and their analogues *ent*-**6** and *ent*-**7** appeared to be able to increase NAGLU protein levels (Figure 8b). Furthermore, *in vitro* enzymatic assays (Figure 8c) showed that the active iminosugars enhance NAGLU enzymatic activity while not acting as NAGLU inhibitors. Co-localization of NAGLU with lysosomal associated membrane protein 1 by confocal immunofluorescence microscopy revealed the ability of l-iminosugars to properly sort the mutated NAGLU enzyme toward the lysosomal compartment. These results suggest that our compounds are able to reduce the lysosomal phenotype of Sanfilippo cellular models possibly by acting as nonactive site pharmacological chaperones.

Moreover, treatment with the active l-iminosugars of epithelial cancer HeLa cells, which contain an elevated amount of HS proteoglycans on the cell surface,^72^ and a nonmutated NAGLU, caused a reduction of HS, resulting in a decreased cell proliferation. These findings are consistent with the known role of HS chains as key modulators of cancer cell proliferation due to their interaction with growth factors and their receptors, leading to overstimulation of downstream signalings.^73^ The active iminosugars appeared not only to act by modulating the expression and/or activity of NAGLU enzyme which catalyzes the fifth step of HS breakdown, but they also seemed to affect HS biosynthetic pathway. Our investigation demonstrated that only *ent*-**1** and *ent*-**2**, the unnatural enantiomers of **1** and **2**, respectively, reduced the expression levels of EXT1 and EXT2, while the other two active iminosugars *ent*-**6** and *ent*-**7** had an opposite effect. These results suggest that these compounds act through a different mechanism affecting the activity of other enzymes involved in the complex HS biosynthetic machinery that still need to be clearly understood. Indeed, EXT1 or EXT2 are members of the exostosin (EXT) family of glycosyltransferases which catalyze the elongation of HS chains through the alternate addition of glucuronic acid and *N*-acetylglucosamine to the HS backbone.

However, HS-controlled biosynthesis involves additional enzymatic reactions (*N*-deacetylation and *N*-sulfation of glucosamine, C-5 epimerization of glucuronic acid to iduronic acid, 2- and 3-*O*-sulfation of uronic acid and glucosamine, respectively, and 6-*O*-sulfation of *N*-acetylated or *N*-sulfated glucosamine residues), as well as posttranslational modifications, which account for the enormous structural and functional heterogeneity of HS chains.^74^

An overview of the results obtained in this study is reported in Table 1.

**Table 1:**
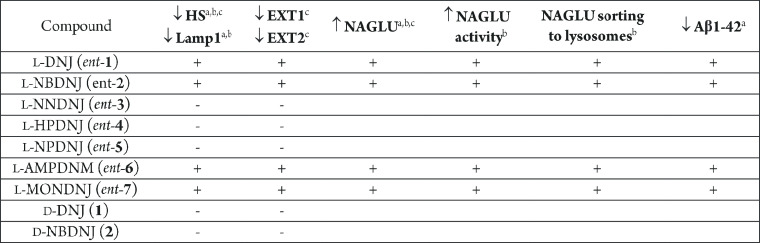
Biological Activity of l-Iminosugars in Sanfilippo B Cellular Models and HeLa Cells

a in ΔNAGLU (MPS IIIB) clone.

b in MPS IIIB fibroblasts.

c in HeLa cells.

Overall, our findings enabled the identification of four l-iminosugars which, after being further validated through both *in vitro* and *in vivo* tests, could contribute to the development of an effective treatment of the clinical manifestation of Sanfilippo syndrome. It is worth recalling that some of these iminosugars have been already found to hold other important pharmacological properties, and these results further augment their value in drug discovery and more generally the importance of l-iminosugars, as they can now be regarded as broad-spectrum pharmacological tools rather than academic curiosities. Herein, in preliminary *in vitro* models, our compounds have displayed highly promising properties, demonstrating to hit the target even by more than a single mechanism of action. In addition, differently from most iminosugar drug candidates reported to date, the lack of inhibition of these l-iminosugars toward carbohydrate-processing enzymes enables association of high efficacy with selectivity and safety properties which have always limited the development of iminosugars in drug discovery.

## Experimental Section

### Chemical Synthesis

#### General Information

All chemicals and solvents were used at the highest degree of purity and without further purification (Sigma-Aldrich, Alfa Aesar, VWR). Thin-layer chromatography analysis was carried out to follow the reaction course by using F254 Merck silica gel plates and subsequent exposure to ultraviolet radiation, iodine vapor, and spraying with ethanolic *p*-anisaldehyde solution. Intermediates and final products were purified by column chromatography with silica gel (70–230 mesh, Merck Kiesegel 60) and characterized by NMR analysis (NMR spectrometers: Varian Inova 500 MHz and Bruker AVANCE 400 MHz). Matrix-assisted laser desorption/ionization (MALDI) mass spectrometry (MS) analysis was performed with an AB SCIEX TOF/TOF 5800 MALDI mass spectrometer working in high-resolution reflectron mode. Optical rotations were measured at 25 ± 2 °C in the stated solvent. Iminosugars *ent*-**2**–**6** were synthesized as previously reported.^39,40^ All compounds were herein converted into the corresponding hydrochloride salt by addition of 1 M HCl (1.0 equiv) followed by evaporation of volatiles. Absolute quantitative nuclear magnetic resonance (qNMR) experiments were performed to assess the purity of compounds following the “general guidelines for quantitative 1D ^1^H NMR (qHNMR) experiments”, provided by the *Journal of Medicinal Chemistry*. In all cases, purity was ≥95% (see the Supporting Information for details).

#### Allyl 2,3,4,6-Tetra-*O*-benzyl-l-glucopyranoside (**12**)

To a stirred solution of l-glucose (**11**, 5.0 g, 28.0 mmol) in allyl alcohol (150 mL), BF_3_OEt_2_ (1.0 mL, 8.1 mmol) was added, and the resulting suspension was warmed to the reflux temperature. After 16 h, the mixture was cooled to rt, neutralized by addition of triethylamine, and volatiles were removed under reduced pressure. The resulting residue was diluted with anhydrous DMF (90 mL), and NaH (60% dispersion in oil mineral, 4.6 g, 190 mmol) was added at 0 °C. BnBr (16 mL, 140 mmol) was added dropwise, and the reaction mixture was slowly warmed to rt and stirred for 2 h at the same temperature. Afterward, the reaction mixture was quenched with aq NH_4_Cl and extracted with EtOAc. The combined organic layers were washed with brine, dried (Na_2_SO_4_), and evaporated under reduced pressure. Chromatography of the crude residue over silica gel (hexane/EtOAc = 9:1) gave the pure allyl 2,3,4,6-tetra-*O*-benzyl-l-glucopyranoside (**12**, 14.6 g, 91% overall yield from l-glucose). NMR data were fully in agreement with those reported elsewhere for the corresponding d-enantiomer.^75^

#### 2,3,4,6-Tetra-*O*-benzyl-l-glucopyranose (**13**)

PdCl_2_ (0.85 g, 4.8 mmol) was added to a stirred solution of allyl 2,3,4,6-tetra-*O*-benzyl-l-glucopyranoside (**12**, 14.0 g, 24.0 mmol) in MeOH (135 mL). The reaction mixture was stirred for 16 h at rt, then filtered on a Celite pad, and washed with Et_2_O. The filtrate was evaporated under reduced pressure and purified by chromatography over silica gel (hexane/EtOAc = 7:3) to provide the pure 2,3,4,6-tetra-*O*-benzyl-l-glucopyranose (**13**, 12.3 g, 95% yield) whose ^1^H and ^13^C NMR spectra were fully in agreement with those reported in literature for its d-enantiomer.^75^

#### 2,3,4,6-Tetra-*O*-benzyl-l-deoxynojirimycin (**15**)

*Step i*: LiAlH_4_ (1.69 g, 44.0 mmol) was slowly added to a stirred solution of **13** (12.0 g, 22.0 mmol) in anhydrous THF (126 mL) at 0 °C and under an argon atmosphere. The reaction mixture was stirred at rt for 20 h and then H_2_O and EtOAc were added until achieving neutral pH. The resulting suspension was extracted with EtOAc and washed with brine. The organic layers were dried (Na_2_SO_4_) and concentrated under reduced pressure. The resulting glucitol (12.0 g, 22.0 mmol) was used in the oxidation step without further purification. *Step ii*: a solution of DMSO (7.9 mL, 110.0 mmol) in DCM (66.0 mL) was slowly added to a stirred solution of (CO)_2_Cl_2_ (7.1 mL, 84.0 mmol) in DCM (76.0 mL) at −78 °C under an argon atmosphere. After 30 min, a solution of 2,3,4,6-tetra-*O*-benzyl-l-glucitol (12.0 g, 22.0 mmol) in DCM (60 mL) was slowly added dropwise. The resulting solution was stirred for 2 h at −78 °C and then triethylamine (40 mL, 290 mmol) was added. The reaction mixture was kept at the same temperature and stirred for further 3 h and then warmed to rt to obtain a crude residue containing intermediate **14**. The residue obtained was added to a stirred suspension of NH_4_OAc (19.0 g, 24.0 mmol), Na_2_SO_4_ (15.0 g, 10.0 mmol), and NaBH_3_CN (5.5 g, 88.0 mmol) in MeOH (1.1 L) at 0 °C. The suspension was stirred for 20 h at rt. NaOH was then added until pH = 10, and the mixture was extracted with DCM and washed with brine. The organic layers were dried (Na_2_SO_4_), concentrated under reduced pressure, and chromatographed over silica gel (hexane/EtOAc = 6:4) to give the pure 2,3,4,6-tetra-*O*-benzyl-l-deoxynojirimycin (**15**, 8.6 g, 75% overall yield). ^1^H and ^13^C NMR spectra were fully in agreement with those reported in the literature for its d-enantiomer.^46^

#### l-Deoxynojirimycin·HCl (*ent*-**1**)

BCl_3_ (1 M solution in DCM, 57 mL, 57.0 mmol) was added to a stirred solution of **15** (8.5 g, 16.0 mmol) in DCM (0.43 L) at 0 °C. The mixture was stirred for 12 h at the same temperature and then quenched with MeOH (0.35 L) at 0 °C and concentrated under reduced pressure. The crude residue was triturated with EtOAc to give pure l-DNJ·HCl (*ent*-**1**) (3.0 g, 92% yield). NMR data was consistent with the NMR spectra reported elsewhere.^39^^1^H NMR (400 MHz, D_2_O) δ: 3.03 (dd, 1H, *J* = 11.6, 12.6 Hz), 3.26 (ddd, 1H, *J* = 3.2, 5.2, 10.5 Hz), 3.56 (dd, 1H, *J* = 5.4, 12.6 Hz), 3.65 (dd, 1H, *J* = 9.3, 10.5 Hz), 3.77–3.87 (m, 2H), 3.93 (dd, 1H, *J* = 5.2, 12.8 Hz), 4.00 (dd, 1H, *J* = 3.2, 12.8 Hz). ^13^C NMR (100 MHz, D_2_O) δ: 41.5, 53.6, 55.8, 62.8, 63.7, 72.2. [α]_D_ −44.0, *c* 0.43, MeOH. MALDI-TOF MS: *m*/*z* 163.0845 (calcd); 164.1478 [M + H]^+^, 186.1443 [M + Na]^+^ (found). Purity was ≥95%, as determined by qNMR.

#### l-*N*-Butyl DNJ HCl (*ent*-**2**)^39^

^1^H NMR (400 MHz, CD_3_OD) δ: 1.01 (t, 3H, *J* = 7.3 Hz), 1.38–1.51 (m, 2H), 1.65–1.85 (m, 2H), 2.99 (t, 1H, *J* = 11.8 Hz), 3.05 (bd, 1H, *J* = 9.8 Hz), 3.20 (td, 1H, *J* = 5.0, 12.6 Hz), 3.33–3.42 (m, 2H), 3.45 (dd, 1H, *J* = 4.8, 11.8 Hz), 3.60 (t, 1H, *J* = 9.8 Hz), 3.67–3.73 (m, 1H), 3.91 (d, 1H, *J* = 12.4 Hz), 4.12 (d, 1H, *J* = 12.4 Hz). ^13^C NMR (100 MHz, D_2_O) δ: 12.9, 19.6, 24.7, 48.9, 52.4, 53.8, 55.2, 65.2, 67.1, 68.3, 76.8. [α]_D_ +5.6, *c* 0.41, H_2_O. MALDI-TOF MS: *m*/*z* 219.1471 (calcd); 220.2204 [M + H]^+^, 242.2182 [M + Na]^+^, 258.1969 [M + K]^+^ (found). Purity was ≥95%, as determined by qNMR.

#### *N*-Nonyl-l-DNJ HCl (*ent*-**3**)^40^

^1^H NMR (400 MHz, CD_3_OD) δ: 0.84 (t, 3H, *J* = 7.0 Hz), 1.19–1.40 (m, 12H), 1.60–1.80 (m, 2H), 2.94 (t, 1H, *J* = 10.7 Hz), 3.01 (d, 1H, *J* = 9.3 Hz), 3.14 (td, 1H, *J* = 5.4, 11.7 Hz), 3.21–3.27 (m, 1H), 3.33 (t, 1H, *J* = 9.3 Hz), 3.40 (dd, 1H, *J* = 4.8, 12.0 Hz), 3.55 (t, 1H, *J* = 9.3 Hz), 3.66 (ddd, 1H, *J* = 5.0, 9.3, 10.7 Hz), 3.85 (dd, 1H, *J* = 3.0, 12.3 Hz), 4.05 (d, 1H, *J* = 12.3 Hz). ^13^C NMR (100 MHz, CD_3_OD) δ: 14.4, 23.7, 24.2, 27.7, 30.2, 30.3, 30.5, 33.0, 54.4, 54.8, 55.0, 67.5, 67.8, 68.9, 78.1. [α]_D_ +9.0, *c* 0.62, MeOH. MALDI-TOF MS: *m*/*z* 289.2253 (calcd); 290.3331 [M + H]^+^, 312.3356 [M + K]^+^ (found). Purity was ≥95%, as determined by qNMR.

#### *N*-[5-(Hexoxy)pentyl]-l-DNJ HCl (*ent*-**4**)^40^

^1^H NMR (400 MHz, CD_3_OD) δ: 0.92 (t, 3H, *J* = 6.2 Hz), 1.24–1.42 (m, 6H), 1.45–1.53 (m, 2H), 1.54–1.61 (m, 2H), 1.62–1.71 (m, 2H), 1.78–1.90 (m, 2H), 3.01 (dd, 1H, *J* = 11.6, 12.0 Hz), 3.06 (bd, 1H, *J* = 10.2 Hz), 3.17–3.27 (m, 1H), 3.35–3.41 (m, 2H), 3.42–3.52 (m, 5H), 3.61 (dd, 1H, *J* = 9.0, 10.2 Hz), 3.67–3.74 (m, 1H), 3.92 (d, 1H, *J* = 12.4 Hz), 4.14 (d, 1H, *J* = 12.4 Hz). ^13^C NMR (100 MHz, CD_3_OD) δ: 14.4, 23.7, 23.9, 24.5, 27.0, 30.1, 30.7, 33.0, 54.3, 54.9, 67.4, 67.8, 68.8, 71.4, 72.1, 78.1. [α]_D_ +4.2, *c* 0.57, MeOH. MALDI-TOF MS: *m*/*z* 333.2515 (calcd); 334.3745 [M + H]^+^ (found). Purity was ≥95%, as obtained by qNMR.

#### *N*-[5-(Nonyloxy)pentyl]-l-DNJ HCl (*ent*-**5**)^40^

^1^H NMR (400 MHz, CD_3_OD) δ: 0.89 (t, 3H, *J* = 6.2 Hz), 1.21–1.39 (m, 12H), 1.40–1.49 (m, 2H), 1.50–1.59 (m, 2H), 1.60–1.69 (m, 2H), 1.70–1.87 (m, 1H), 2.98 (t, 1H, *J* = 11.6 Hz), 3.04 (bd, 1H, *J* = 9.6 Hz), 3.14–3.25 (m, 1H), 3.32–3.39 (m, 2H), 3.40–3.48 (m, 5H), 3.59 (dd, 1H, *J* = 9.6, 10.4 Hz), 3.68–3 (ddd, 1H, *J* = 4.8, 9.3, 11.6 Hz), 3.89 (dd, 1H, *J* = 2.9, 12.4 Hz), 4.10 (d, 1H, *J* = 12.4 Hz). ^13^C NMR (100 MHz, CD_3_OD) δ: 14.4, 23.7, 23.9, 24.5, 27.3, 30.1, 30.4, 30.6, 30.7, 30.8, 33.0, 54.3, 54.9, 55.0, 67.4, 67.8, 68.8, 71.4, 72.1, 78.2. [α]_D_ +10.0, *c* 0.53, MeOH. MALDI-TOF MS: *m*/*z* 375.2985 (calcd); 376.4492 [M + H]^+^, 398.4445 [M + Na]^+^ (found). Purity was ≥95%, as determined by qNMR.

#### *N*-Adamantanemethoxypentyl-l-DNJ (*ent*-**6**)^40^

^1^H NMR (500 MHz, CD_3_OD) δ: 1.47–1.57 (m, 2H), 1.58–1.62 (m, 6H), 1.65–1.75 (m, 5H), 1.76–1.93 (m, 5H), 2.0 (br s, 3H), 3.01–3.06 (m, 3H), 3.08 (bd, 1H, *J* = 10.9 Hz), 3.25 (td, 1H, *J* = 4.4, 12.4 Hz), 3.37–3.52 (m, 5H), 3.64 (t, 1H, *J* = 10.9 Hz), 3.68–3.76 (m, 1H), 3.94 (d, 1H, *J* = 12.3 Hz), 4.16 (d, 1H, *J* = 12.3 Hz). ^13^C NMR (125 MHz, CD_3_OD) δ: 23.9, 24.6, 29.7, 30.1, 35.1, 38.3, 40.9, 54.4, 54.9, 67.4, 67.8, 68.8, 72.1, 78.2, 83.1. [α]_D_ +9.0, *c* 0.68, H_2_O. MALDI-TOF MS: *m*/*z* 397.2828 (calcd); 398.4402 [M + H]^+^, 420.4393 [M + Na]^+^ (found). Purity was ≥95%, as determined by qNMR.

#### 1,9-Diiodononane (**17**)

Iodine (5.2 g, 20.4 mmol) was added to a stirring solution of polymer-supported triphenylphosphine (PS-TPP; 100–200 mesh, extent of labeling: ∼3 mmol/g triphenylphosphine loading) (6.8 g, 21.0 mmol) in anhydrous DCM (52 mL) under an argon atmosphere. After 10 min, 1,9-nonanediol (**16**, 0.82 g, 5.12 mmol) was added to the slight yellow suspension, and the reaction was stirred at rt for 1 h. Afterward, the suspension was filtered with DCM to remove triphenylphosphine oxide and washed with saturated Na_2_S_2_O_3_ and brine and extracted with DCM. Organic layers were dried (Na_2_SO_4_) and evaporated under reduced pressure, affording the desired 1,9-diiodononane as a pale-yellow oil (**17**, 1.8 g, 95% yield). ^1^H NMR (400 MHz, CDCl_3_): δ 1.21–1.47 (m, 10H), 1.76–1.88 (m, 4H), 3.19 (t, 4H, *J* = 7.0 Hz); ^13^C NMR (100 MHz, CDCl_3_): ppm 7.3, 28.4, 29.2, 30.4, 33.5. MALDI-TOF MS: *m*/*z* 379.9498 (calcd); 402.5157 [M + Na]^+^ (found). Purity was ≥95%, as determined by qNMR.

#### 1-Iodo-9-methoxynonane (**18**)

NaH (60% dispersion in mineral oil, 0.20 g, 5.1 mmol) was added to a stirred solution of methanol (0.24 mL, 5.9 mmol) in anhydrous THF (7.5 mL) at 0 °C and under an argon atmosphere. The reaction mixture was stirred at the same temperature for 1 h, then a solution of bis-iodide **17** (1.5 g, 3.9 mmol) in THF (7.5 mL) was added. The solution was warmed to rt and stirred for 48 h. Afterward, DCM was added, and the solution was washed with aq NH_4_Cl and brine. The organic layer was dried with Na_2_SO_4_ and the solvent evaporated under reduced pressure. Chromatography of the crude residue over silica gel (hexane/EtOAc = 95:5) gave the pure iodide **18** (8, 0.83 g, 75% yield) as oil. ^1^H NMR (400 MHz, CDCl_3_): δ 1.21–1.47 (m, 8H), 1.51–1.63 (m, 4H), 1.76–1.88 (m, 2H), 3.19 (t, 2H, *J* = 7.0 Hz), 3.30 (s, 3H), 3.36 (t, 2H, *J* = 6.6 Hz); ^13^C NMR (125 MHz, CHCl_3_): ppm 7.3, 26.1, 28.5, 29.3, 29.4, 29.6, 30.4, 33.5, 58.5, 72.9. MALDI-TOF MS: *m*/*z* 284.0637 (calcd); 326.4973 [M + ACN + H]^+^ (found). Purity was ≥95% by qNMR.

#### *N*-(9′-Methoxynonyl)-l-DNJ (*ent*-**7**)

To a stirred solution of *ent*-**1** (0.10 g, 0.61 mmol) in anhydrous DMF (2 mL), K_2_CO_3_ (0.25 g, 1.8 mmol) was added at rt under an argon atmosphere. A solution of iodide **18** (0.21 g, 0.73 mmol) in DMF (2 mL) was added dropwise and then the reaction mixture was warmed to 80 °C and stirred for 16 h. The solvent was then removed under reduced pressure and chromatographed over silica gel (acetone/MeOH = 8:2) to give the pure l-MONDNJ, which was then converted into the corresponding hydrochloride salt by addition of 1 M HCl (0.61 mmol) followed by evaporation of volatiles (0.15 g, 75% yield). ^1^H NMR (500 MHz, CD_3_OD): δ 1.28–1.49 (m, 10H), 1.52–1.63 (m, 2H), 1.67–1.88 (m, 2H), 2.95–3.11 (m, 2H), 3.13–3.27 (m, 1H), 3.31 (s, 3H), 3.40 (t, 4H, *J* = 6.5 Hz), 3.47 (dd, 1H, *J* = 4.9, 11.8 Hz), 3.61 (t, *J* = 11.8 Hz, 1H), 3.65–3.77 (m, 1H), 3.91 (d, 1H, *J* = 11.6 Hz), 4.14 (d, 1H, *J* = 11.6 Hz); ^13^C NMR (125 MHz, CD_3_OD): ppm 24.2, 27.1, 27.6, 30.1, 30.4, 30.5, 30.6, 54.4, 54.8, 54.9, 58.7, 67.4, 67.8, 68.8, 73.9, 78.2. [α]_D_ +7.7, *c* 0.87, MeOH. MALDI-TOF MS: *m*/*z* 319.2359 (calcd); 320.3464 [M + H]^+^, 342.3463 [M + Na]^+^ (found). Purity was ≥95%, as determined by qNMR.

## Biological Evaluation

### Materials

Dulbecco’s modified Eagle’s medium (DMEM), RPMI-1640, fetal bovine serum (FBS), penicillin, streptomycin, and phosphate-buffered saline (PBS) were provided by Thermo Fisher Scientific (Carlsbad, CA, USA); formaldehyde solution 37% (F15587), methanol (34860), Trizma base (T1503), glycine (G8898), acrylamide/bis-acrylamide 30% solution (A3699) were provided by Sigma-Aldrich (St. Louis, MI, USA); ProLong Gold Antifade Mountant with DAPI (P36935) was obtained from Thermo Fisher Scientific (Carlsbad, CA, USA); ECL system was obtained from Amersham Pharmacia (Buckinghamshire, UK); Bradford assay reagent was purchased from Bio-Rad (München, Germany); bovine serum albumin (BSA) (10711454001) and protease inhibitor cocktail tablets (04693132001) were obtained from Roche Diagnostics (Grenoble, France).

### Antibodies

The following antibodies were used for immunofluorescence analyses: mouse monoclonal anti-CD107a (anti-Lamp1) (SAB4700416 clone H4A3) from Sigma-Aldrich (St. Louis, MI, USA), rabbit polyclonal anti-GM130 (#12480) from Cell Signaling Technology (Danvers, MA, USA), mouse monoclonal anti-HS (clone 10E4) from AMSBIO, rabbit anti-NAGLU monoclonal antibody (ab214671) and rabbit monoclonal anti-β-Amyloid (Aβ) 1-42 antibody [mOC64] (ab201060) from Abcam (Cambridge, MA, USA), and mouse and rabbit Alexa-Fluor (488 and 546) secondary antibodies A11029, A11030, A11034, and A11035 from Thermo Fisher Scientific-Invitrogen (Carlsbad, CA, USA). For Western blot analysis, rabbit anti-NAGLU monoclonal antibody (ab214671) from Abcam (Cambridge, MA, USA), mouse anti-β-actin monoclonal antibody (13E5) from Cell Signaling Technology (Danvers, Massachusetts), rabbit anti-EXT1 polyclonal antibody (D01P) from Abnova (Taipei City, Taiwan), rabbit anti-EXT2 polyclonal antibody (11348-1-AP) from Proteintech (Deansgate, Manchester), mouse anti-SDC2 polyclonal antibody (B04P) from Abnova (Taipei City, Taiwan), and mouse anti-GAPDH monoclonal antibody (6C5 sc-32233) from Santa Cruz Biotechnology (Heidelberg, Germany) were used; secondary antibodies used were goat anti-mouse IgG polyclonal antibody conjugated to horseradish peroxidase (HRP) (sc-2031) and goat anti-rabbit IgG-HRP polyclonal antibody (sc-3837) from Santa Cruz Biotechnology (Heidelberg, Germany).

### Cell Culture

Stable NAGLU-silenced clones were obtained from SK-NBE human neuroblastoma cell line (CRL-2271 ATCC, Wesel, Germany) as previously described.^45^ SK-NBE neuroblastoma stable clones were cultured in RPMI-1640, 2 mM l-glutamine, 1 mM sodium pyruvate, supplemented with 10% FBS, 100 units/mL penicillin, 100 μg/mL streptomycin, and 0.7 μg/mL of puromycin at 37 °C in a humidified 5% CO_2_ atmosphere.

Fibroblasts from MPS-affected patients were kindly provided by the Cell Line and DNA Biobank from Patients Affected by Genetic Diseases (Istituto G. Gaslini, Genoa, Italy).^50^ Primary human dermal fibroblasts, adult (HDFa, from Thermo Fisher Scientific) and MPS fibroblasts were cultured in DMEM, supplemented with 10% FBS, 2 mM l-glutamine, 100 U/mL penicillin, and 100 mg/mL streptomycin, at 37 °C in a humidified 5% CO_2_ atmosphere.

HeLa cells were cultured in DMEM, supplemented with 10% FBS, 2 mM l-glutamine, 100 U/mL penicillin, and 100 mg/mL streptomycin, at 37 °C in a humidified 5% CO_2_ atmosphere.

### Proliferation Assay

HeLa cells were seeded in 24-well plates at a concentration of 100,000 cells per well and grown overnight in complete RPMI medium.^76−78^ HeLa cells were incubated with 20 μM of each selected l- and d-iminosugar diluted in the same medium. Control HeLa cells (mock) were untreated. After 48 h of incubation at 37 °C, the cells were trypsinized, and the number of alive cells, resuspended in a solution of trypan blue, was determined by direct counts by using a BioRad automatized cell count system. The data reported are the means of three independent experiments performed with each sample in replicates of three. Error bars indicate standard errors of the means (s.e.m). Student’s *t*-test was used for statistical comparisons, and differences were considered significant at *p* < 0.05.

### Fluorescence Microscopy

Immunofluorescence staining was performed as previously reported.^79−82^ Briefly, cells grown on glass coverslips were washed with PBS and fixed in 3.7% formaldehyde at room temperature for 30 min. After fixation, cells were washed with PBS and permeabilized by incubation in blocking buffer (PBS containing 1% BSA, 0.01% sodium azide, and 0.02% saponin) for 20 min at room temperature. Cells were then incubated with the indicated primary antibodies diluted in the same blocking buffer for 1 h at room temperature. Cells were washed three times with PBS and incubated with the corresponding secondary antibodies for 30 min at room temperature. Finally, coverslips were washed in distilled water and mounted onto glass slides with the Prolong Gold anti-fade reagent with DAPI. Images were collected using a laser-scanning microscope (LSM 700, Carl Zeiss Microimaging, Inc., Jena, Germany) equipped with a Plan Apo 63× oil immersion (NA 1.4) objective lens.

### Western Blotting

Cells grown to subconfluence in standard medium were harvested in lysis buffer (50 mM Tris pH 7.5, 150 mM NaCl, 1 mM EDTA, 1 mM EGTA, 10% glycerol, 1% Triton X-100, 1 mM β-glycerophosphate, 1 mM phenylmethylsulfonyl fluoride, protease inhibitor cocktail tablet, 1 mM sodium orthovanadate, and 2.5 mM sodium pyrophosphate).^83−85^ The lysates were incubated for 30 min on ice, and supernatants were collected and centrifuged for 30 min at 14,000*g*. Protein concentration was estimated by Bradford assay, and 50 μg/lane of total proteins were separated on SDS gels and transferred to nitrocellulose membranes. Membranes were treated with a blocking buffer (25 mM Tris, pH 7.4, 200 mM NaCl, 0.5% Triton X-100) containing 5% nonfat powdered milk for 1 h at room temperature.^86−88^ Incubation with the primary antibody was carried out overnight at 4 °C. After washings, membranes were incubated with the HRP-conjugated secondary antibody for 1 h at room temperature. Following further washings of the membranes, chemiluminescence was generated by an enhanced chemiluminescence (ECL) kit.

### Enzyme Activity Assay

To determine NAGLU enzymatic activity in HDFa and MPS IIIB fibroblasts, pellets of 5 × 10^5^ cells were collected, subjected to 10 freeze–thaw cycles, and clarified by centrifugation at 13 rpm for 30 min at 4 °C. Protein concentration of samples was determined by the Lowry method. NAGLU enzymatic activity was measured as described by Marsh and Fensom using 4-methylumbelliferyl-*N*-acetyl-α-d-glucosaminide as the fluorogenic substrate.^54^ 25 μL cell lysates (HDFa and MPS IIIB) were incubated for 2 h with 50 μL of substrate solution (2 mM) and 25 μL of 0.2 M Na-acetate buffer pH 4.5. *ent*-**1**, *ent*-**2**, *ent*-**6**, and *ent*-**7** iminosugars were added to the reaction mix of the MPS IIIB lysate at the concentration of 20 μM each. Moreover, to test the inhibitory effects of the active iminosugars toward the nonmutated NAGLU activity, *ent*-**1**, *ent*-**2**, *ent*-**6**, and *ent*-**7** iminosugars were added to the reaction mix of the HDFa lysate at the concentration up to 1 mM. Enzymatic activity was normalized for total protein concentration, and hydrolysis of 1 nmol of substrate per hour per milligram of protein was defined as a catalytic unit.

### Statistical Analysis

Data reported are expressed as the mean ± SD of at least three separate experiments. Statistical significance was determined by Student’s *t*-test and one-way ANOVA test.

## Supporting Information Available

Copies of ^1^H and ^13^C NMR spectra and details for qNMR of synthesized compounds; additional figures concerning biological experiments (PDF)
Molecular formula strings of all synthesized compounds (CSV)

## Abbreviations

AAVs: adeno-associated vectors
AllOH: allyl alcohol
AMPDNM: *N*-adamantanemethoxypentyl deoxynojirimycin
CF: cystic fibrosis
CTRL: control clone
DGJ: deoxygalactonojirimycin
DNJ: deoxynojirimycin
DS: dermatan sulfate
ERT: enzyme replacement therapy
EXT: exostosin glycosyltransferase
GAG: glycosaminoglycan
GNS: *N*-acetylglucosamine-6-sulfate sulfatase
GT: gene therapy
HGSNAT: *N*-acetyltransferase
HPDNJ: *N*-hexyloxypentyl deoxynojirimycin
HS: heparan sulfate
HSCT: hematopoietic stem cell transplantation
Lamp1: lysosomal associated membrane protein 1
LSD: lysosomal storage disease
LVs: lentiviral vectors
MONDNJ: *N*-methoxynonyl deoxynojirimycin
MPS: mucopolysaccharidosis
NBDNJ: *N*-butyl deoxynojirimycin
NNDNJ: *N*-nonyl deoxynojirimycin
NPDNJ: *N*-nonyloxypentyl deoxynojirimycin
PCT: pharmacological chaperone therapy
PrP: prion protein
PS-TPP: polymer-supported triphenylphosphine
SDC2: proteoglycan syndecan 2
SGSH: *N*-sulfoglucosamine sulfohydrolase
SRT: substrate reduction therapy
ΔNAGLU: NAGLU-silenced clone
