# Supplementary material for: N-Substituted l-Iminosugars for the Treatment of Sanfilippo Type B Syndrome
Source: J Med Chem. 2023 Jan 25;66(3):1790–808. doi: 10.1021/acs.jmedchem.2c01617 (PMC9923752; doi:10.1021/acs.jmedchem.2c01617)
Supplement: Supplementary file 1 — jm2c01617_si_001.pdf [file jm2c01617_si_001.pdf]

Supporting Information

N-substituted L-iminosugars for the treatment of Sanfilippo type B syndrome

Valeria De Pasquale,[2,§] Anna Esposito,[3,§] Gianluca Scerra,[1] Melania Scarcella,[1] Mariangela Ciampa,[1] Antonietta Luongo,[4] Daniele D'Alonzo,[5] Annalisa Guaragna,[3,*] Massimo D'Agostino[1,*] and Luigi Michele Pavone[1 *]

[1]Department of Molecular Medicine and Medical Biotechnology, University of Naples Federico II, Via S. Pansini 5, 80131, Naples, Italy; [2]Department of Veterinary Medicine and Animal Productions, University of Naples Federico II, Via F. Delpino 1, 80137, Naples, Italy; [3]Department of Chemical, Materials and Production Engineering, University of Naples Federico II, Piazzale V. Tecchio 80, 80125 Naples, Italy; [4]AORN Sant'Anna e San Sebastiano, via F. Palasciano, 81100, Caserta, Italy; [5]Department of Chemical Sciences, University of Naples Federico II, via Cintia, 80126 Napoli, Italy.

*Correspondence should be addressed to **L.M.P.** (luigimichele.pavone@unina.it), **M.D.A.** (massimo.dagostino@unina.it), and **A.G.** (annalisa.guaragna@unina.it).

[§]These Authors equally contributed to this work.

^{1}H NMR and ^{13}C NMR spectra of L-NBDNJ (*ent*-2) S2

^{1}H NMR and ^{13}C NMR spectra of L-NNDNJ (*ent*-3) S3

^{1}H NMR and ^{13}C NMR spectra of L-HPDNJ (*ent*-4) S4

^{1}H NMR and ^{13}C NMR spectra of L-NPDNJ (*ent*-5) S5

^{1}H NMR and ^{13}C NMR spectra of L-AMPDNM (*ent*-6) S6

^{1}H NMR and ^{13}C NMR spectra of 17 S7

^{1}H NMR and ^{13}C NMR spectra of 18 S8

^{1}H NMR and ^{13}C NMR spectra of L-MONDNJ (*ent*-7) S9

Absolute qNMR data S10

Figure S1 S15

Figure S2 S16

Copies of NMR spectra

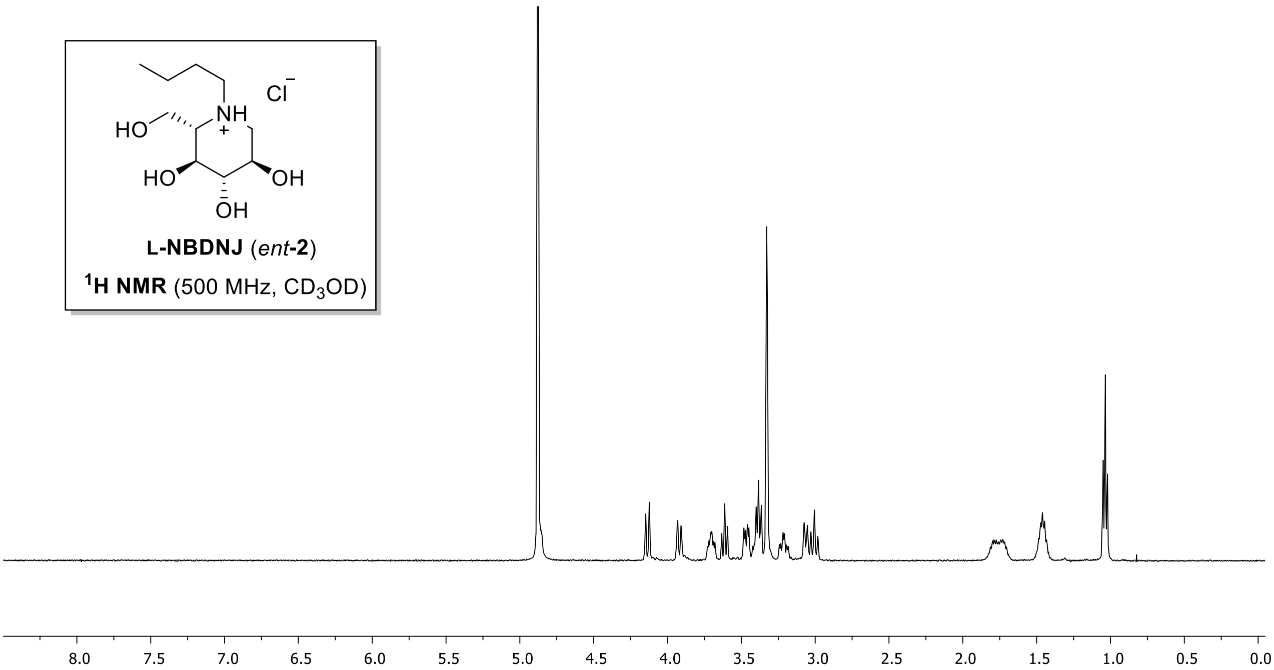

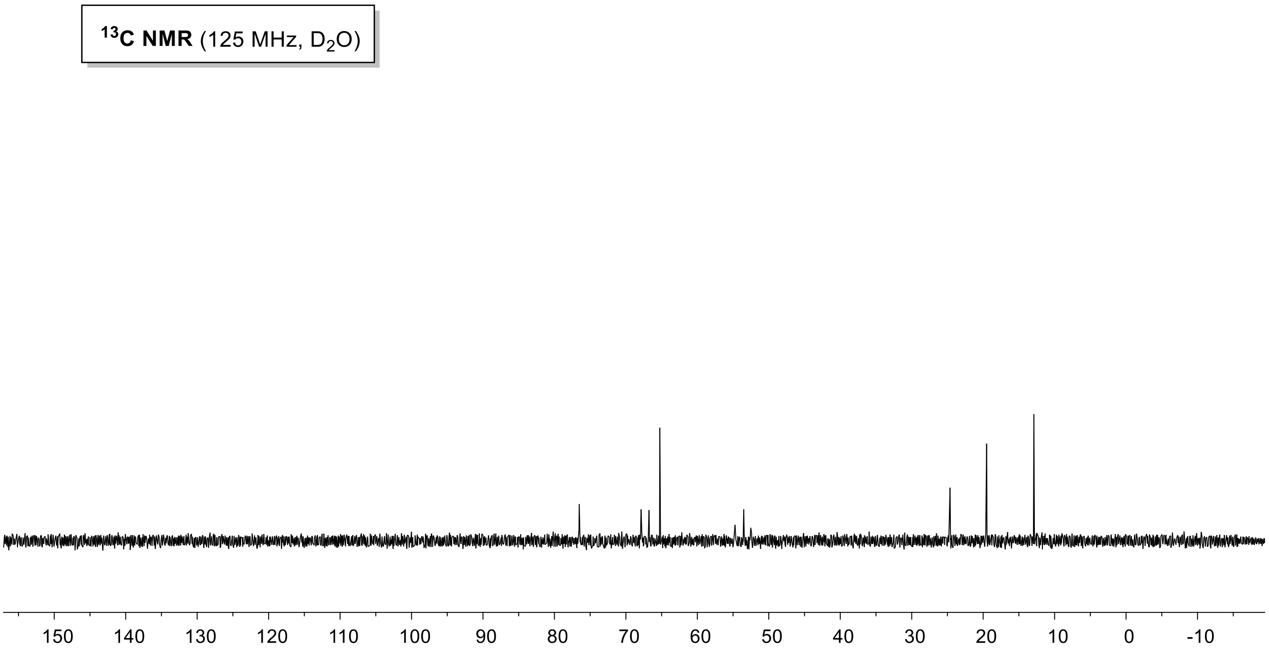

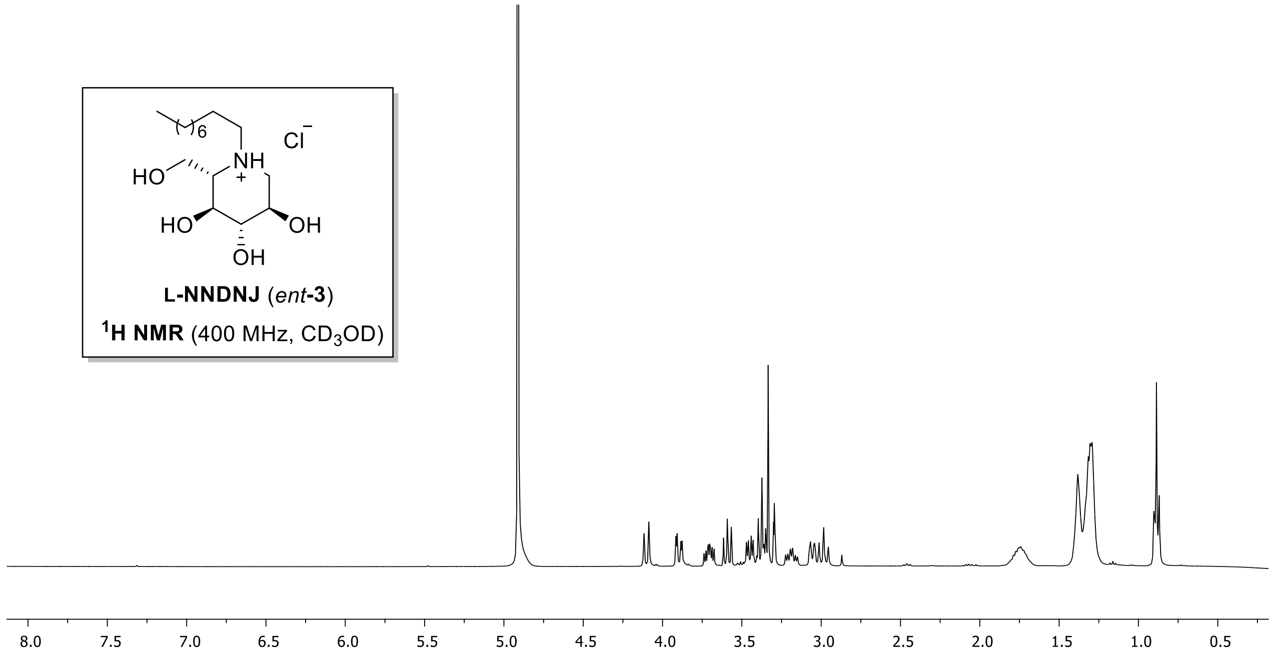
Cl⁻
HO
NH
+
HO
OH
OH
6
L-NNDNJ (ent-3)
1H NMR (400 MHz, CD3OD)

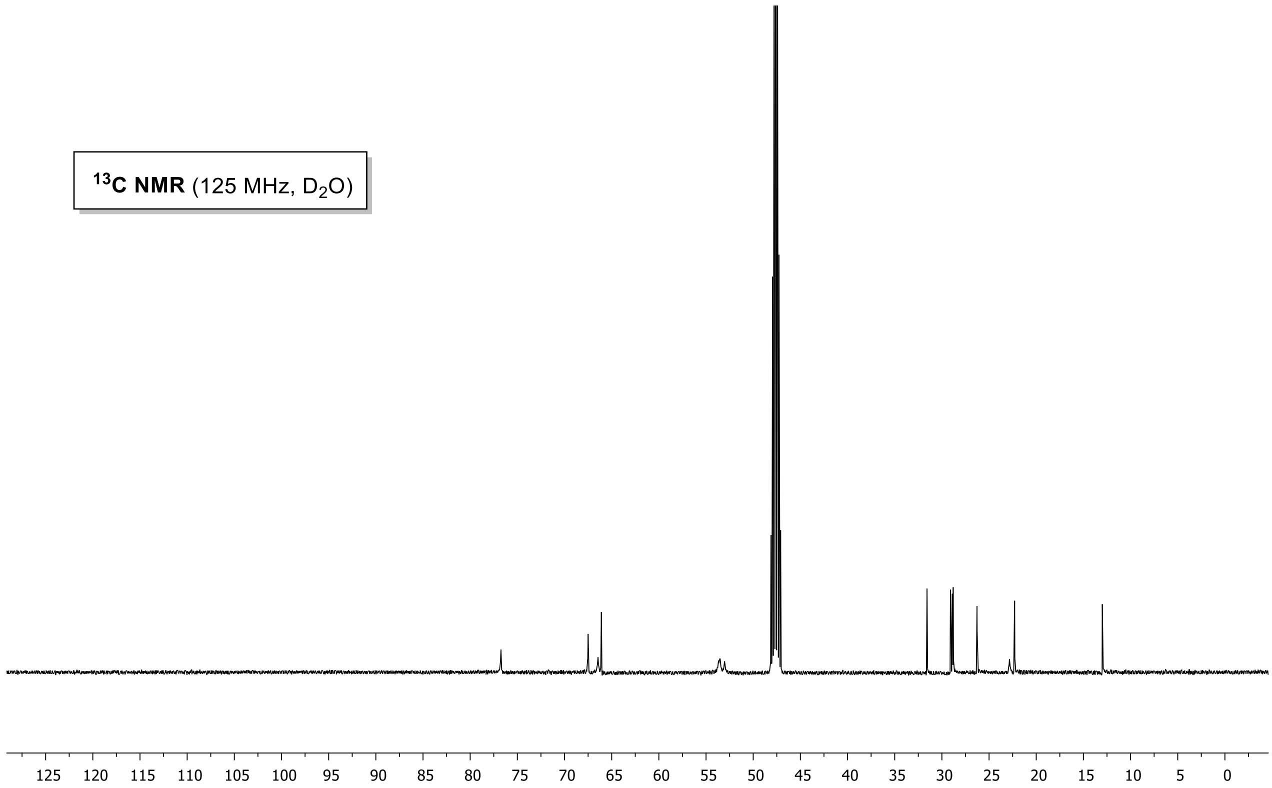
13C NMR (125 MHz, D2O)

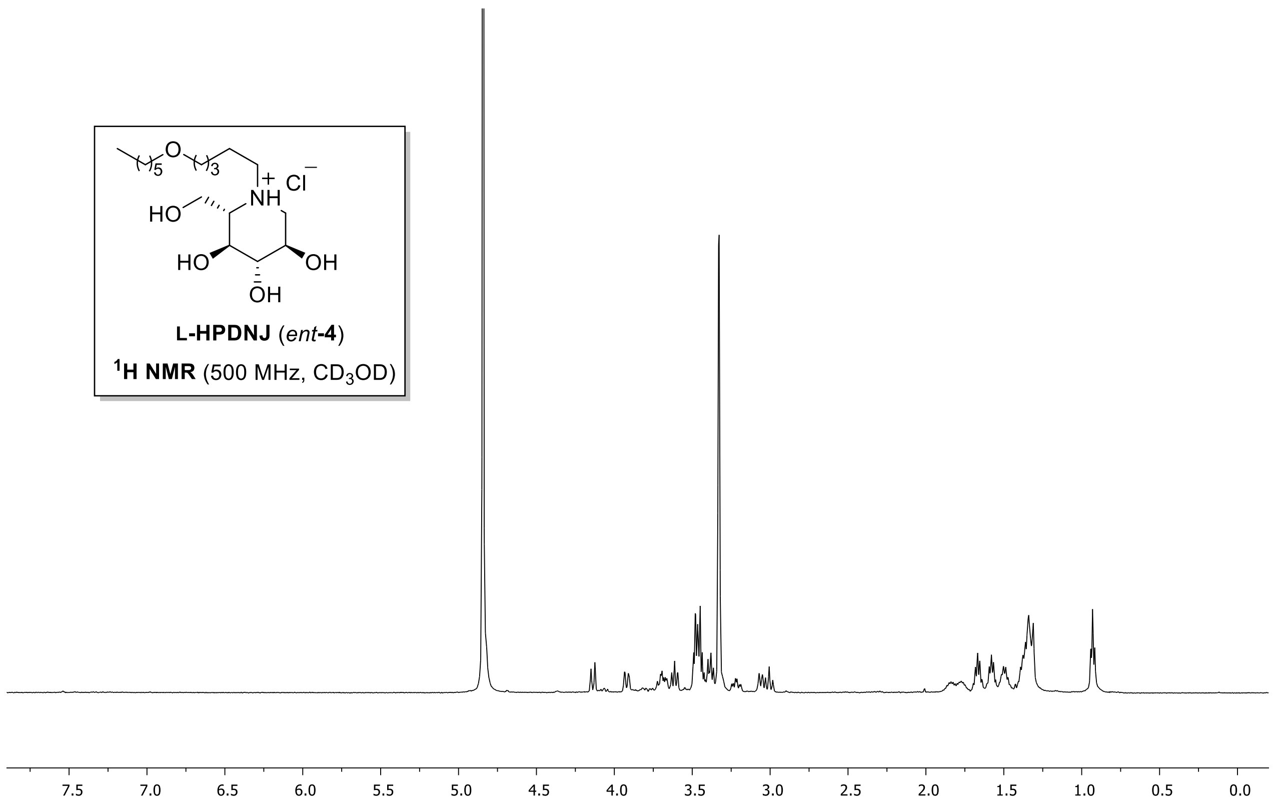
L-HPDNJ (ent-4)
1H NMR (500 MHz, CD3OD)
7.5 7.0 6.5 6.0 5.5 5.0 4.5 4.0 3.5 3.0 2.5 2.0 1.5 1.0 0.5 0.0

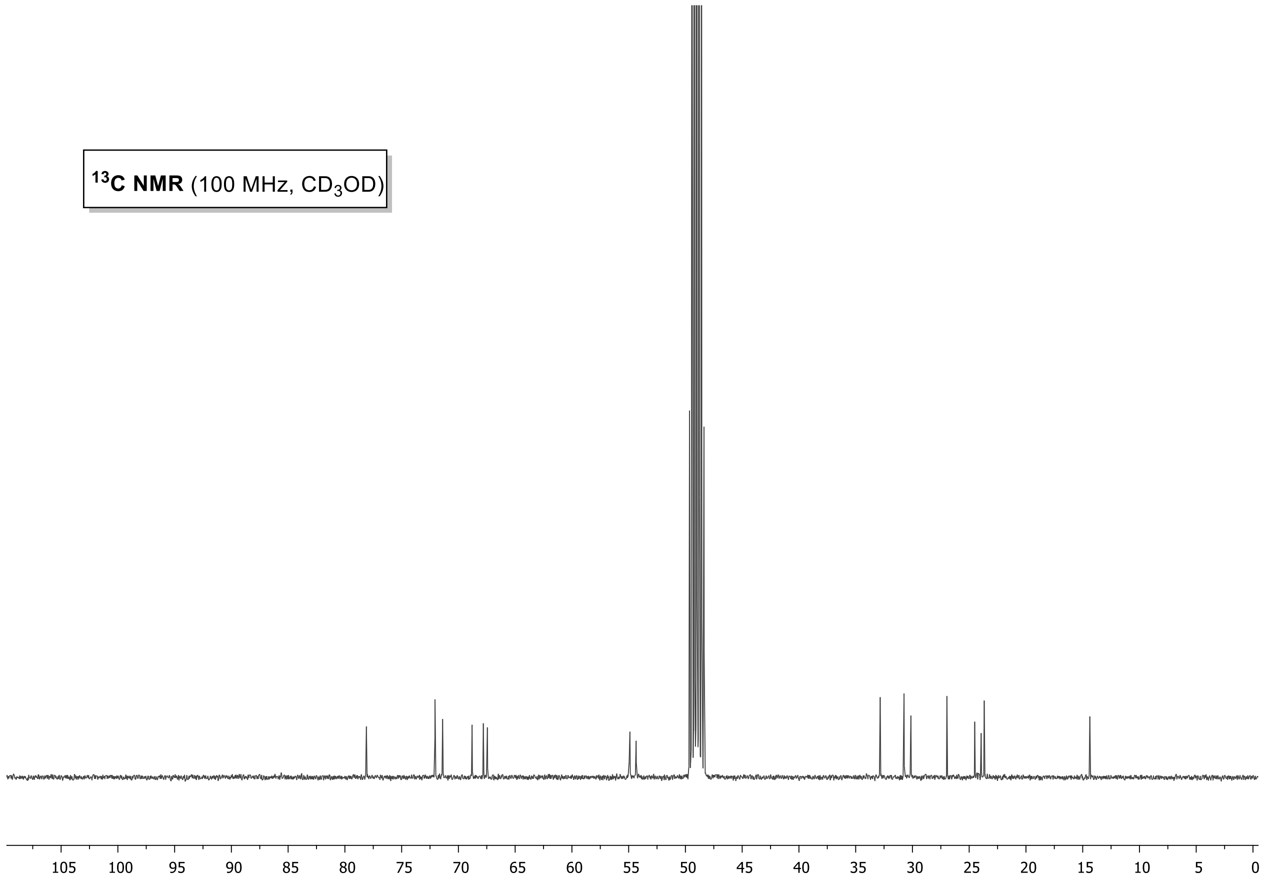
13C NMR (100 MHz, CD3OD)
105 100 95 90 85 80 75 70 65 60 55 50 45 40 35 30 25 20 15 10 5 0

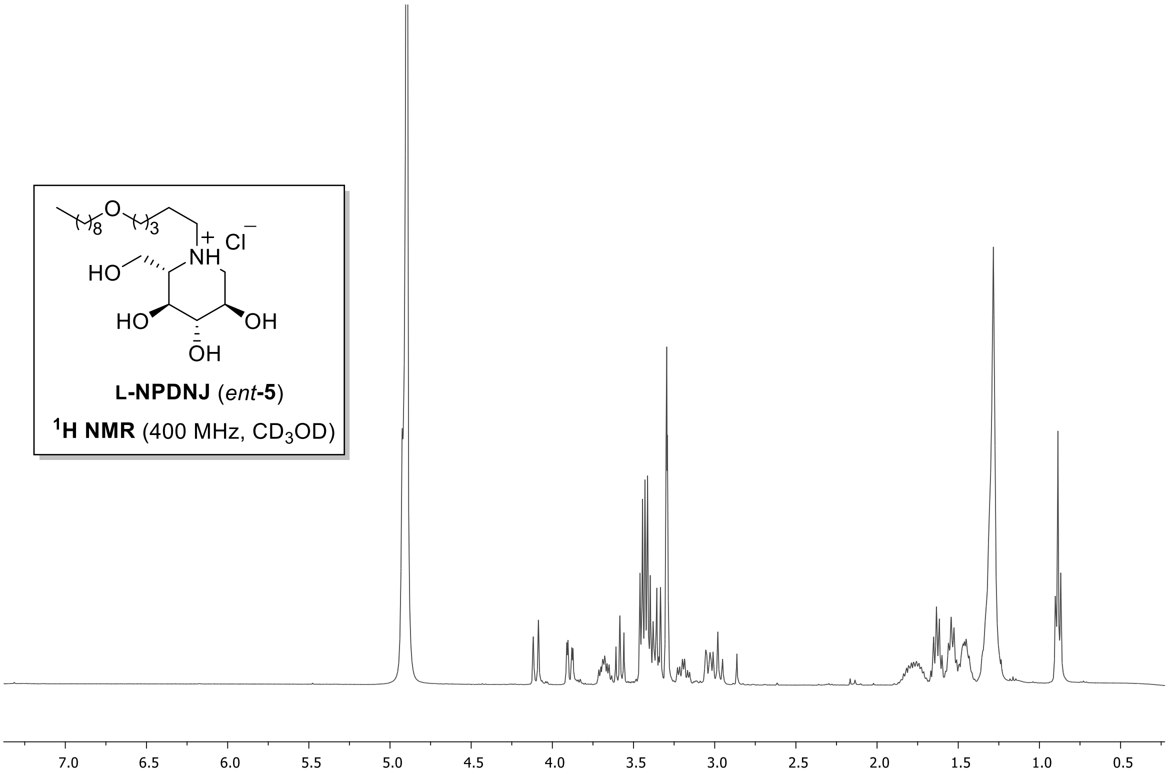
HO
NH
Cl
HO
OH
OH
8
3
O
L-NPDNJ (ent-5)
1H NMR (400 MHz, CD3OD)
7.0
6.5
6.0
5.5
5.0
4.5
4.0
3.5
3.0
2.5
2.0
1.5
1.0
0.5

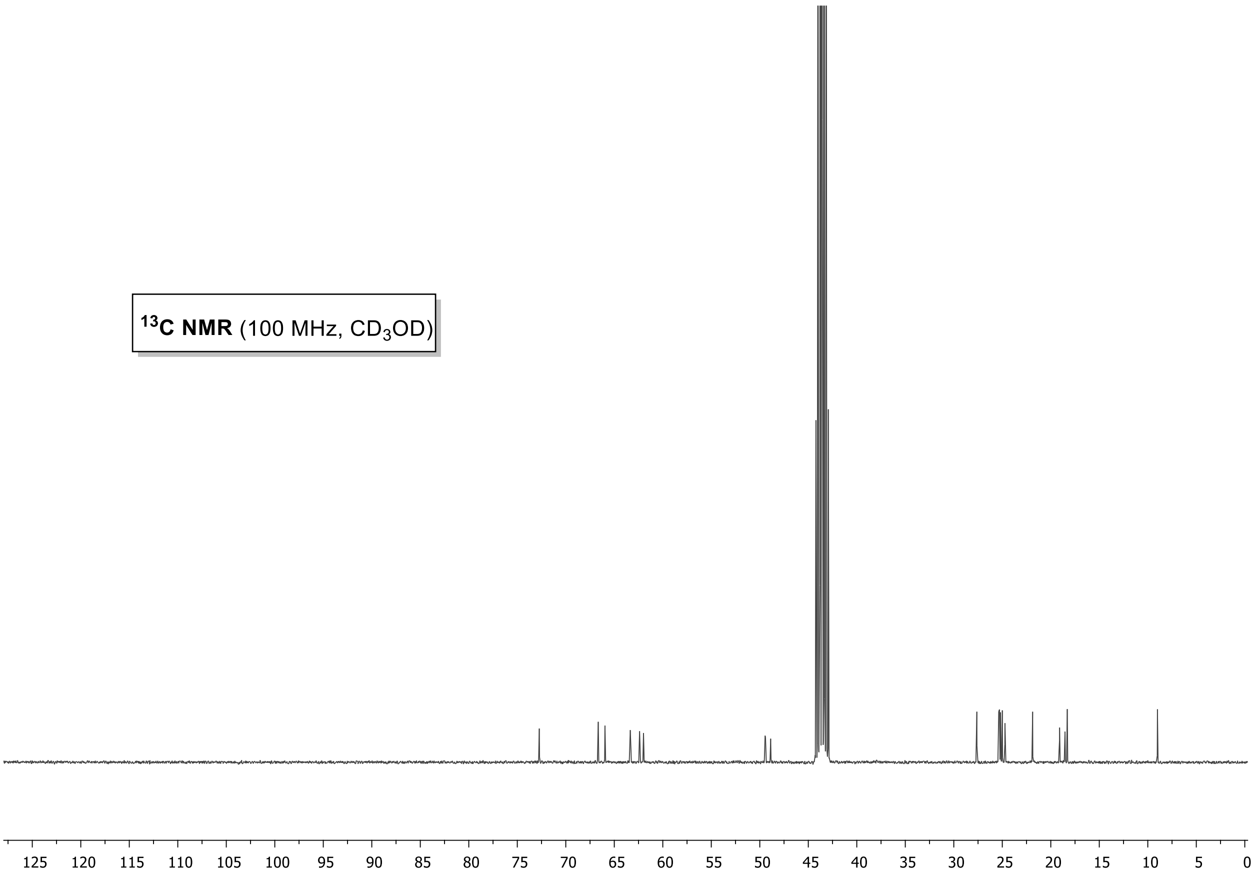
13C NMR (100 MHz, CD3OD)
125
120
115
110
105
100
95
90
85
80
75
70
65
60
55
50
45
40
35
30
25
20
15
10
5
0

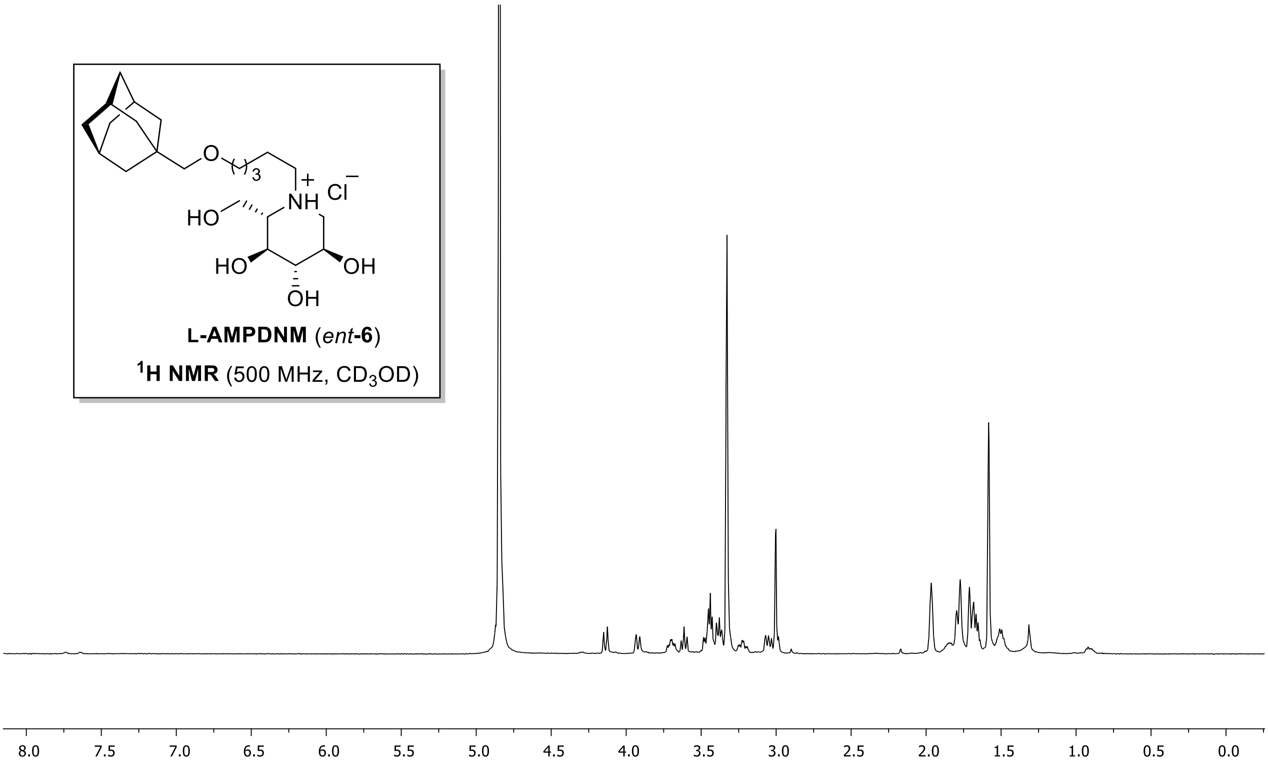
HO
NH
Cl
HO
OH
OH
O
3
L-AMPDNM (*ent*-6)
^{1}H NMR (500 MHz, CD_3OD)
8.0 7.5 7.0 6.5 6.0 5.5 5.0 4.5 4.0 3.5 3.0 2.5 2.0 1.5 1.0 0.5 0.0

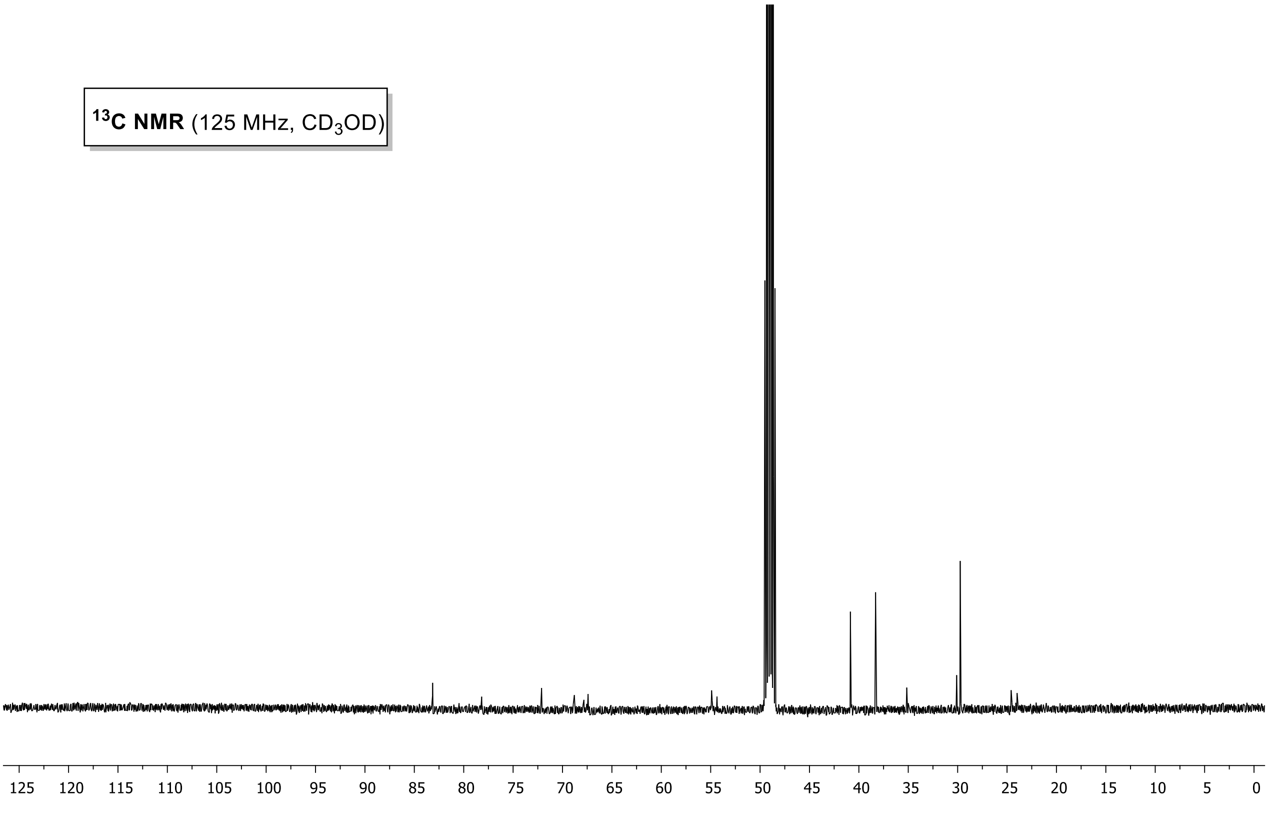
^{13}C NMR (125 MHz, CD_3OD)
125 120 115 110 105 100 95 90 85 80 75 70 65 60 55 50 45 40 35 30 25 20 15 10 5 0

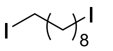

17

^{1}H NMR (400 MHz, $CDCl_3$)

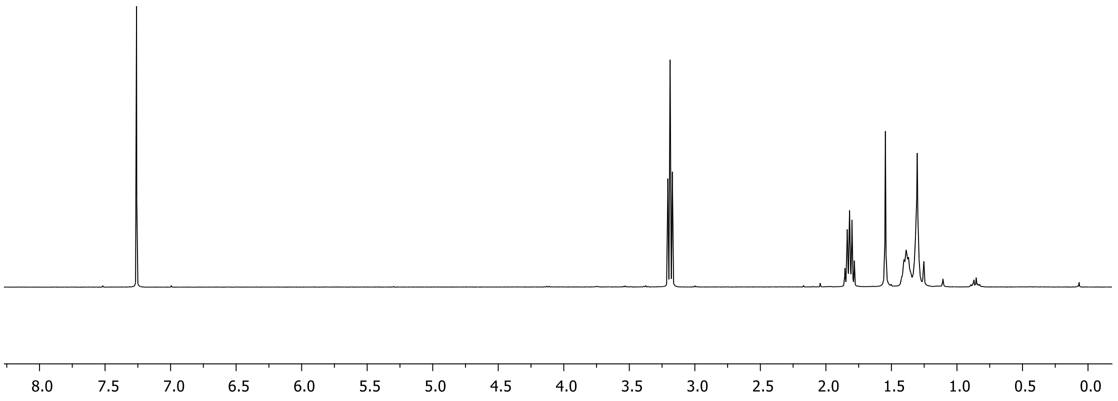

^{13}C NMR (125 MHz, $CDCl_3$)

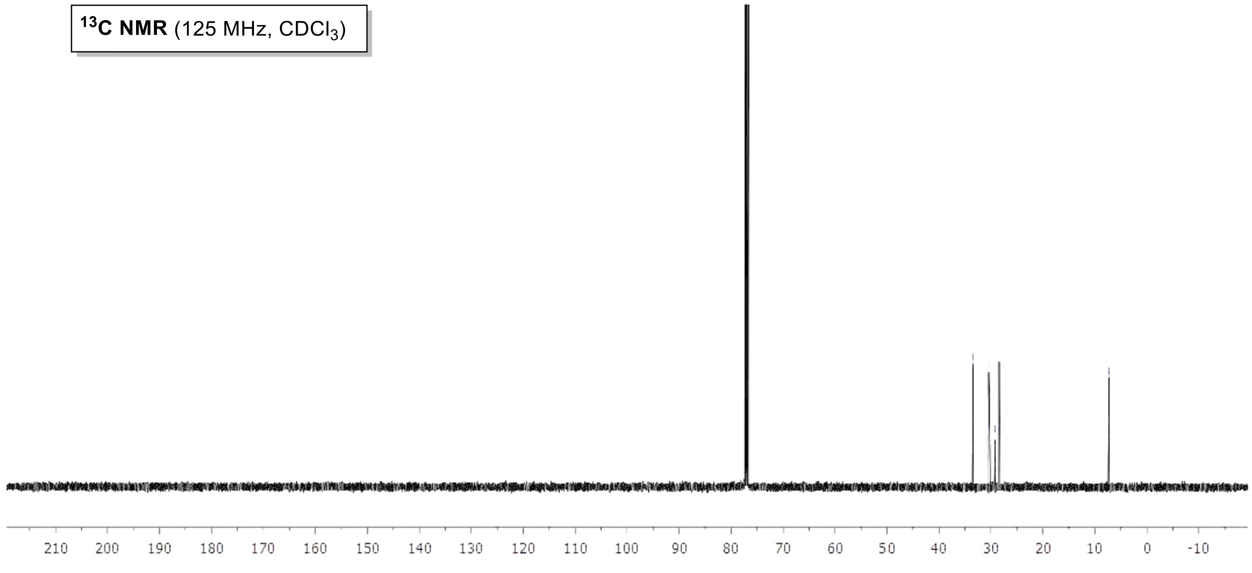

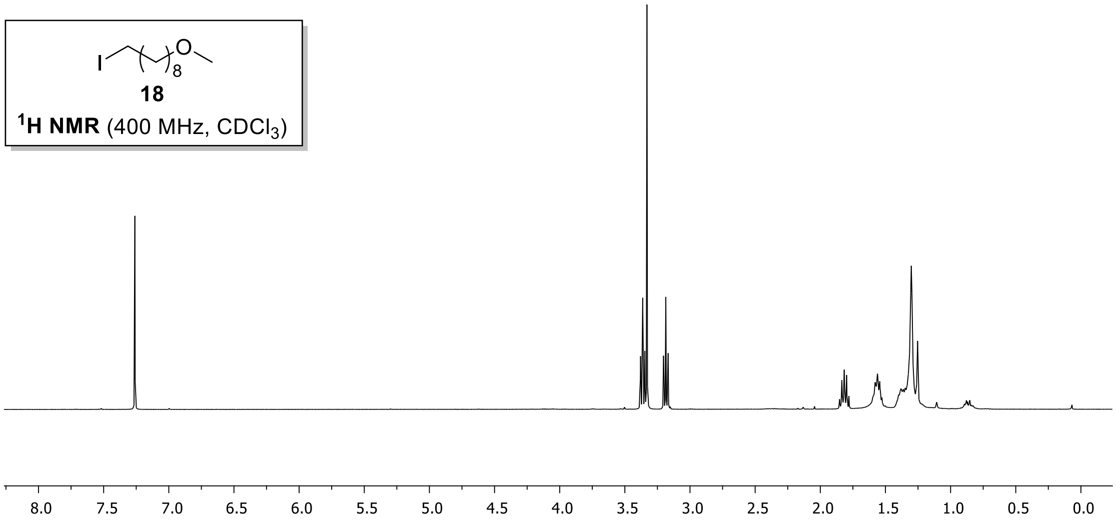
I
O
8
18
^{1}H NMR (400 MHz, $CDCl_3$)
8.0 7.5 7.0 6.5 6.0 5.5 5.0 4.5 4.0 3.5 3.0 2.5 2.0 1.5 1.0 0.5 0.0

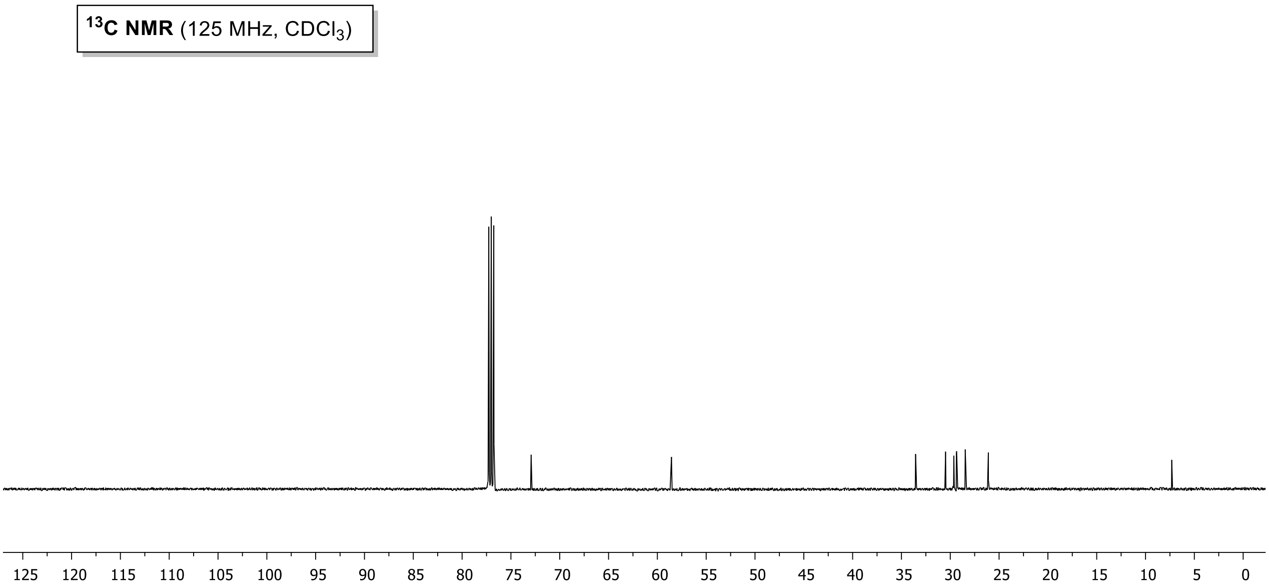
^{13}C NMR (125 MHz, $CDCl_3$)
125 120 115 110 105 100 95 90 85 80 75 70 65 60 55 50 45 40 35 30 25 20 15 10 5 0

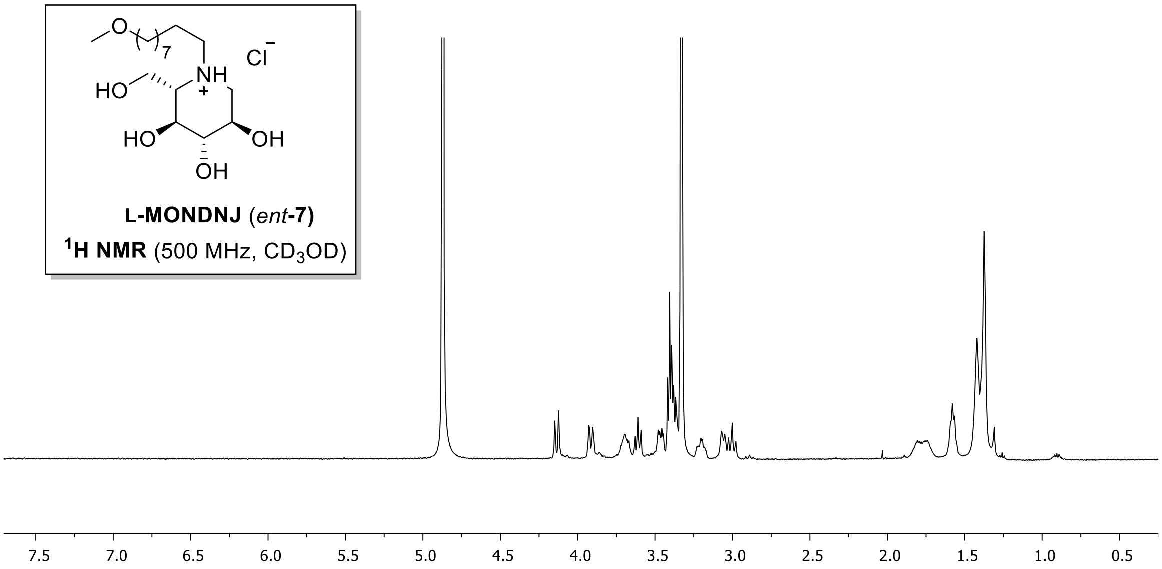
Cl
HO
NH
+
HO
OH
OH
7
O
L-MONDNJ (ent-7)
1H NMR (500 MHz, CD3OD)
7.5 7.0 6.5 6.0 5.5 5.0 4.5 4.0 3.5 3.0 2.5 2.0 1.5 1.0 0.5

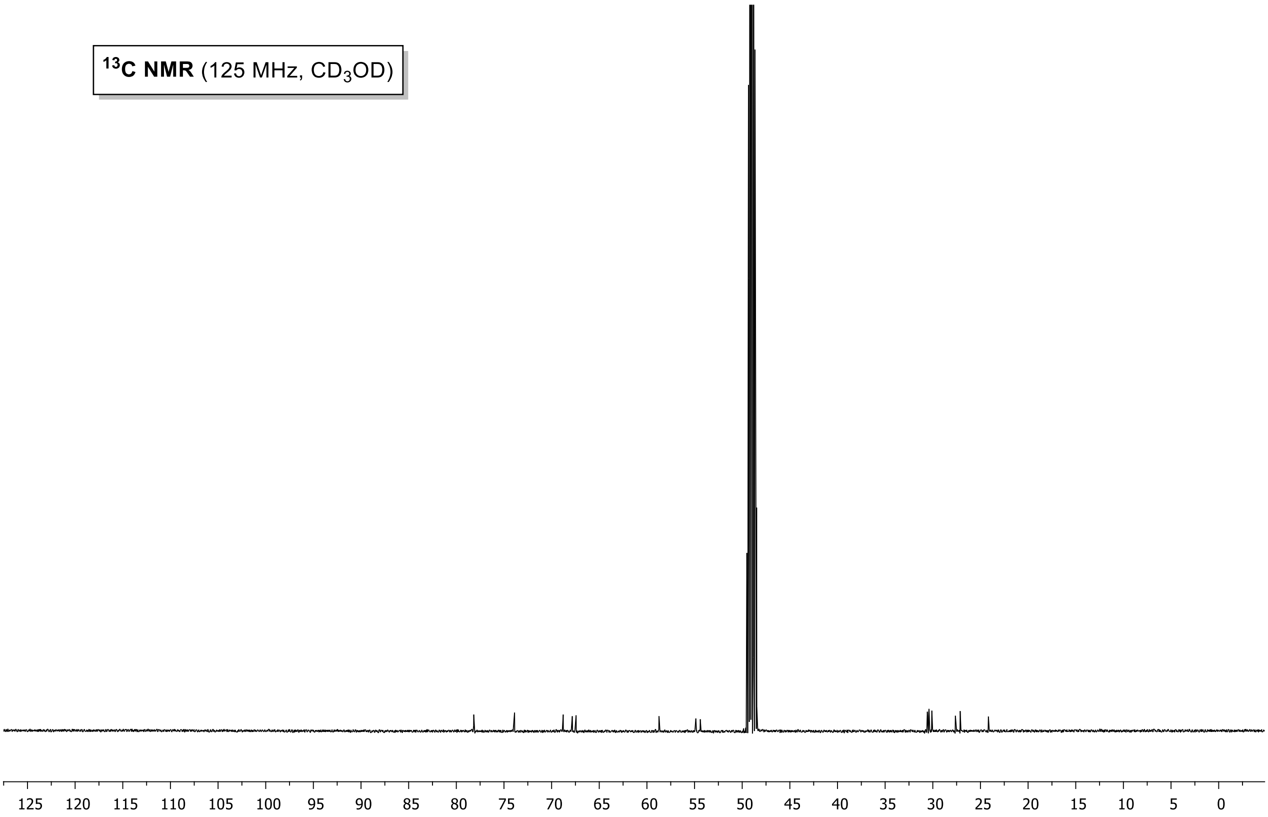
13C NMR (125 MHz, CD3OD)
125 120 115 110 105 100 95 90 85 80 75 70 65 60 55 50 45 40 35 30 25 20 15 10 5 0

Absolute qNMR data

General Procedure.

Absolute qNMR was used for determining of the purity of synthesized compounds following the "general guidelines for quantitative 1D ^{1}H NMR (qHNMR) experiments," provided by the *Journal of Medicinal Chemistry*.

The qHNMR experiments were performed on a Bruker Avance spectrometer (400 MHz) at 298K and benzoic acid was used as internal calibrant (99.5% pure).

Samples and internal standard were weighted into glass vials using an analytical balance with 0.01 mg accuracy and dissolved in CD_3OD or $CDCl_3$ (0.6 mL). The resulting solution was quantitatively transferred into a 5-mm standard NMR tube and spectra were recorded within 2 h after sample preparation. ^{1}H NMR spectra were obtained using "single pulse, zg, with 90° pulse" as pulse program with an acquisition time of 4 s. The number of scans was 64 and width spectral window was 20 ppm. The data were processed with the MestReNova software.

The purity of samples was calculated as follows:

$$\boldsymbol{P}\,[\%] = \frac{\boldsymbol{n_{IC} \cdot Int_t \cdot MW_t \cdot m_{IC}}}{\boldsymbol{n_t \cdot Int_{IC} \cdot MW_{IC} \cdot m_s}}\ \boldsymbol{P_{IC}}$$

Where:
m_{IC} = weight (mass) of the internal calibrant (IC)
m_S = weight mass) of the sample
Int_{IC} = area (integral) of the IC resonance signal being used for quantification
Int_t = area (integral) of the target analyte (t) resonance signal being used for quantification
n_{IC} = number of protons that give rise to Int_{IC}
n_t = number of protons of the target analyte that give rise to Int_t
MW_{IC} = molecular weight of the internal calibrant
MW_t = molecular weight of the target analyte
P_{IC} = purity of the internal calibrant, as percent value

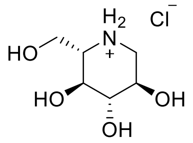

L-DNJ (*ent*-**1**)

^{1}**H NMR** (400 MHz, CD_3OD)

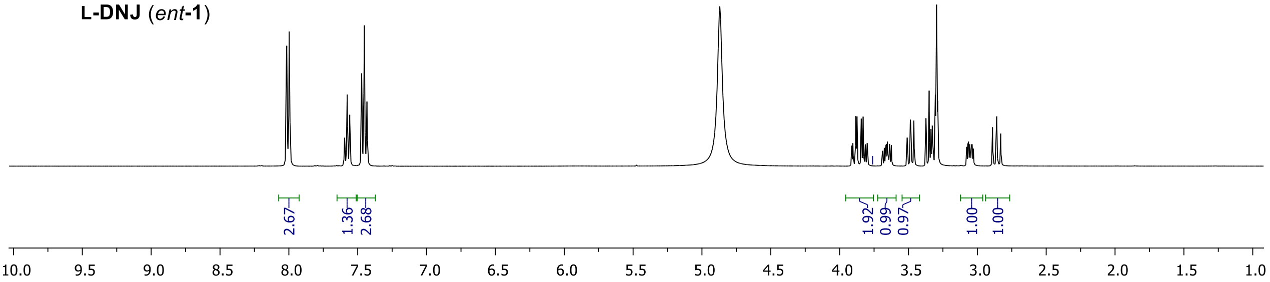

**Step 1**	m_s = 5.71 mg, m_{IC} = 4.73 mg, P_{IC} = 99.5%
**Step 2**	Int_t = 0.98,[a] n_t = 1
**Step 3**	Int_{IC} = 1.34,[b] n_{IC} = 1
**Step 4**	MW_t = 199.63 g/mol, MW_{IC} = 122.12 g/mol
**Step 5**	$P\,[\%] = \frac{n_{IC} \cdot Int_t \cdot MW_t \cdot m_{IC}}{n_t \cdot Int_{IC} \cdot MW_{IC} \cdot m_s}\,P_{IC} = \mathbf{98.5}\%$

[a] The integral of the target analyte was calculated as the average of signals at 2.86, 3.05, 3.49, 3.66 and 3.85 ppm

[b] The integral of the Internal Calibrant was calculated as the average of signals at 7.45, 7.58 and 8.00 ppm

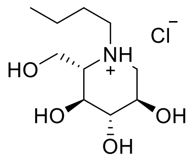

L-NBDNJ (*ent*-**2**)

^{1}**H NMR** (400 MHz, CD_3OD)

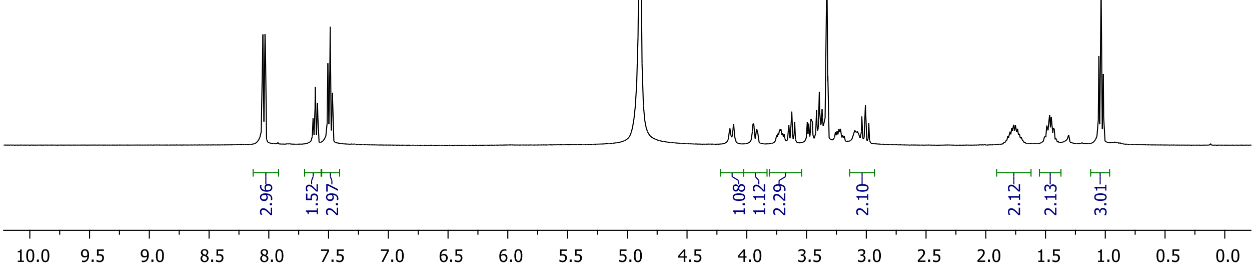

**Step 1**	m_s = 5.42 mg, m_{IC} = 3.53 mg, P_{IC} = 99.5%
**Step 2**	Int_t = 1.06,[a] n_t = 1
**Step 3**	Int_{IC} = 1.49,[b] n_{IC} = 1
**Step 4**	MW_t = 255.74 g/mol, MW_{IC} = 122.12 g/mol
**Step 5**	$P\,[\%] = \frac{n_{IC} \cdot Int_t \cdot MW_t \cdot m_{IC}}{n_t \cdot Int_{IC} \cdot MW_{IC} \cdot m_s}\,P_{IC} = \mathbf{96.5}\%$

[a] The integral of the target analyte was calculated as the average of signals at 1.01, 1.35-1.53, 1.65-1.85, 2.98, 3.07, 3.60, 3.70, 3.90 and 4.10 ppm

[b] The integral of the Internal Calibrant was calculated as the average of signals at 7.45, 7.58 and 8.00 ppm

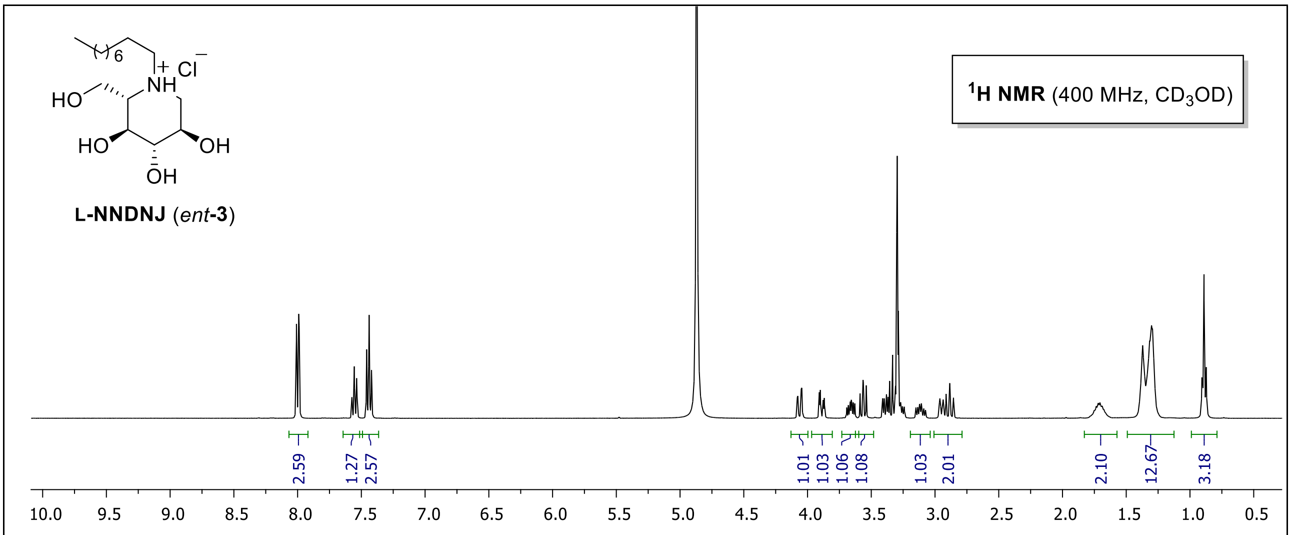

**Step 1**	m_s = 5.47 mg, m_{IC} = 2.55 mg, P_{IC} = 99.5%
**Step 2**	Int_t = 1.04,[a] n_t = 1
**Step 3**	Int_{IC} = 1.29,[b] n_{IC} = 1
**Step 4**	MW_t = 325.87 g/mol, MW_{IC} = 122.12 g/mol
**Step 5**	$P\,[\%] = \frac{n_{IC} \cdot Int_t \cdot MW_t \cdot m_{IC}}{n_t \cdot Int_{IC} \cdot MW_{IC} \cdot m_s}\,P_{IC} = 99.8\%$

[a] The integral of the target analyte was calculated as the average of signals at 0.89, 1.18-1.45, 1.60-1.83, 2.91, 3.11, 3.56, 3.66, 3.88 and 4.06 ppm

[b] The integral of the Internal Calibrant was calculated as the average of signals at 7.45, 7.58 and 8.00 ppm

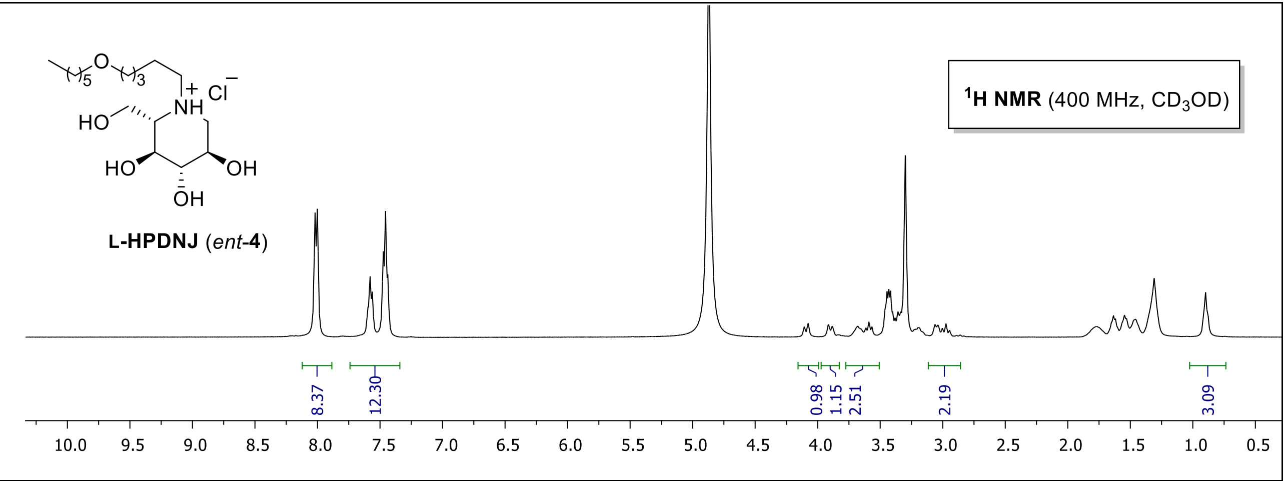

**Step 1**	m_s = 3.21 mg, m_{IC} = 3.87 mg, P_{IC} = 99.5%
**Step 2**	Int_t = 1.10,[a] n_t = 1
**Step 3**	Int_{IC} = 4.13,[b] n_{IC} = 1
**Step 4**	MW_t = 369.93 g/mol, MW_{IC} = 122.12 g/mol
**Step 5**	$P\,[\%] = \frac{n_{IC} \cdot Int_t \cdot MW_t \cdot m_{IC}}{n_t \cdot Int_{IC} \cdot MW_{IC} \cdot m_s}\,P_{IC} = 96.8\%$

[a] The integral of the target analyte was calculated as the average of signals at 0.90, 2.90-3.11, 3.50-3.74, 3.90, and 4.08 ppm

[b] The integral of the Internal Calibrant was calculated as the average of signals at 7.45, 7.58 and 8.00 ppm

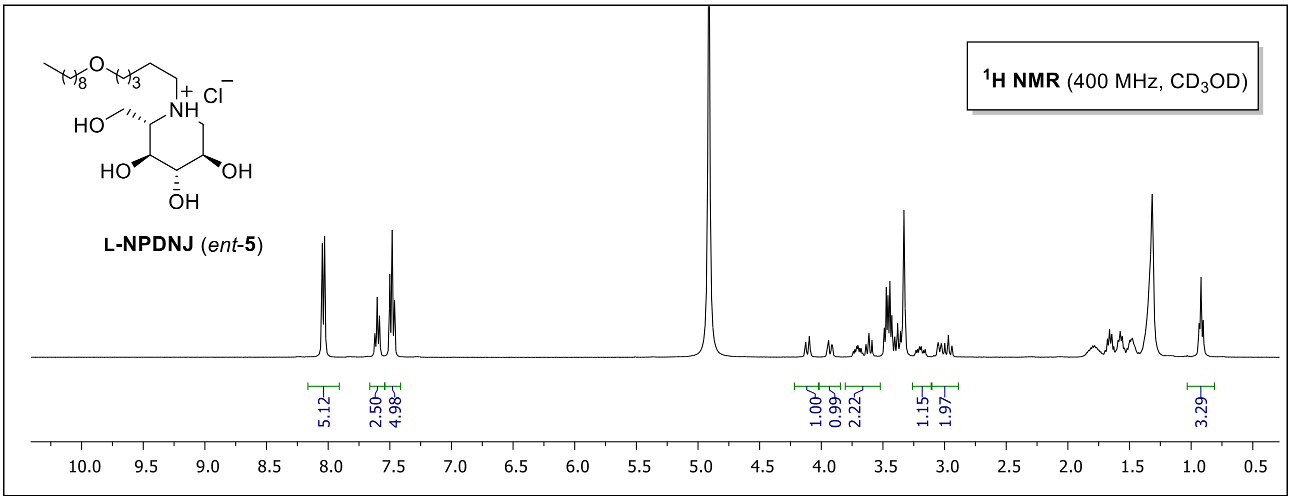

**Step 1**	m_s = 3.68 mg, m_{IC} = 2.59 mg, P_{IC} = 99.5%
**Step 2**	Int_t = 1.06,[a] n_t = 1
**Step 3**	Int_{IC} = 2.52,[b] n_{IC} = 1
**Step 4**	MW_t = 412.01 g/mol, MW_{IC} = 122.12 g/mol
**Step 5**	$P\,[\%] = \frac{n_{IC} \cdot Int_t \cdot MW_t \cdot m_{IC}}{n_t \cdot Int_{IC} \cdot MW_{IC} \cdot m_s}\ P_{IC} = \mathbf{99.4\%}$

[a] The integral of the target analyte was calculated as the average of signals at 0.92, 2.91-3.09, 3.20, 3.54-3.82, 3.92, and 4.10 ppm ppm

[b] The integral of the Internal Calibrant was calculated as the average of signals at 7.45, 7.58 and 8.00 ppm

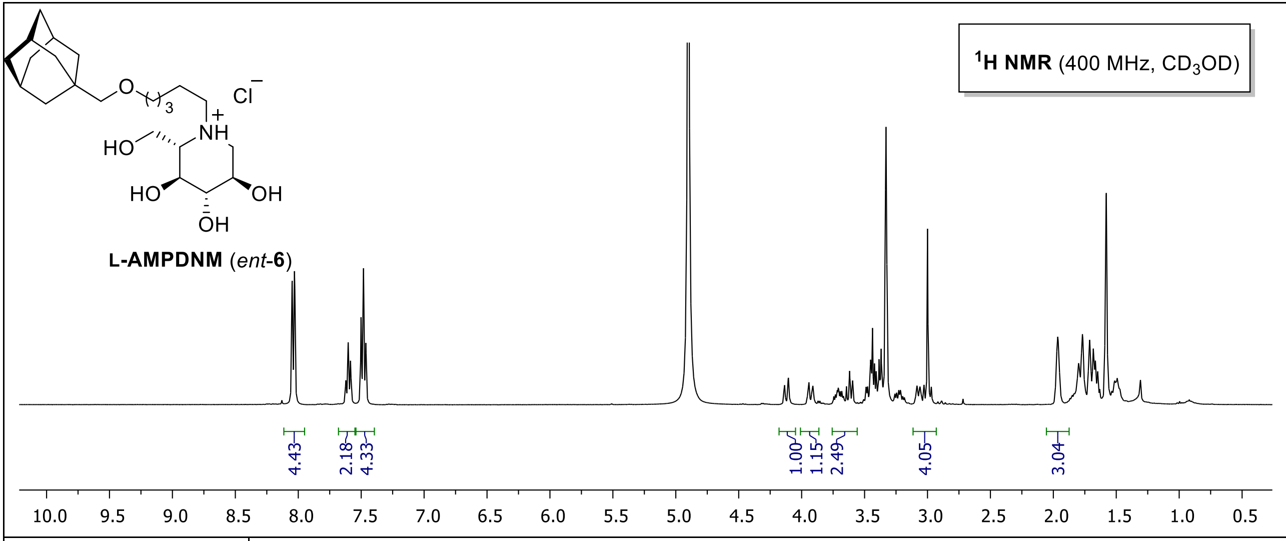

**Step 1**	m_s = 4.54 mg, m_{IC} = 2.54 mg, P_{IC} = 99.5%
**Step 2**	Int_t = 1.07,[a] n_t = 1
**Step 3**	Int_{IC} = 2.19,[b] n_{IC} = 1
**Step 4**	MW_t = 434.01 g/mol, MW_{IC} = 122.12 g/mol
**Step 5**	$P\,[\%] = \frac{n_{IC} \cdot Int_t \cdot MW_t \cdot m_{IC}}{n_t \cdot Int_{IC} \cdot MW_{IC} \cdot m_s}\ P_{IC} = \mathbf{96.7\%}$

[a] The integral of the target analyte was calculated as the average of signals at 1.96, 2.89-3.12, 3.56-3.79, 3.95, and 4.10 ppm

[b] The integral of the Internal Calibrant was calculated as the average of signals at 7.45, 7.58 and 8.00 ppm

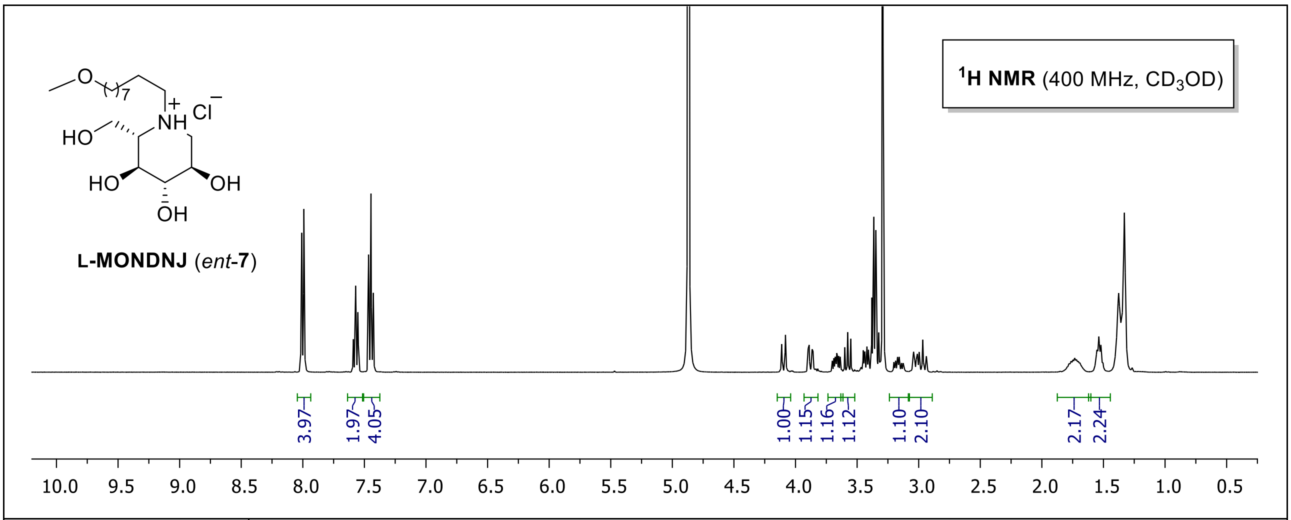

**Step 1**	m_s = 4.53 mg, m_{IC} = 2.85 mg, P_{IC} = 99.5%
**Step 2**	Int_t = 1.09,[a] n_t = 1
**Step 3**	Int_{IC} = 2.00,[b] n_{IC} = 1
**Step 4**	MW_t = 355.90 g/mol, MW_{IC} = 122.12 g/mol
**Step 5**	$P\,[\%] = \frac{n_{IC}\cdot Int_t\cdot MW_t\cdot m_{IC}}{n_t\cdot Int_{IC}\cdot MW_{IC}\cdot m_s}\,P_{IC} = \mathbf{99.4\%}$

[a] The integral of the target analyte was calculated as the average of signals at 1.55, 1.73, 2.90-3.10, 3.19, 3.58, 3.66, 3.89 and 4.10 ppm

[b] The integral of the Internal Calibrant was calculated as the average of signals at 7.45, 7.58 and 8.00 ppm

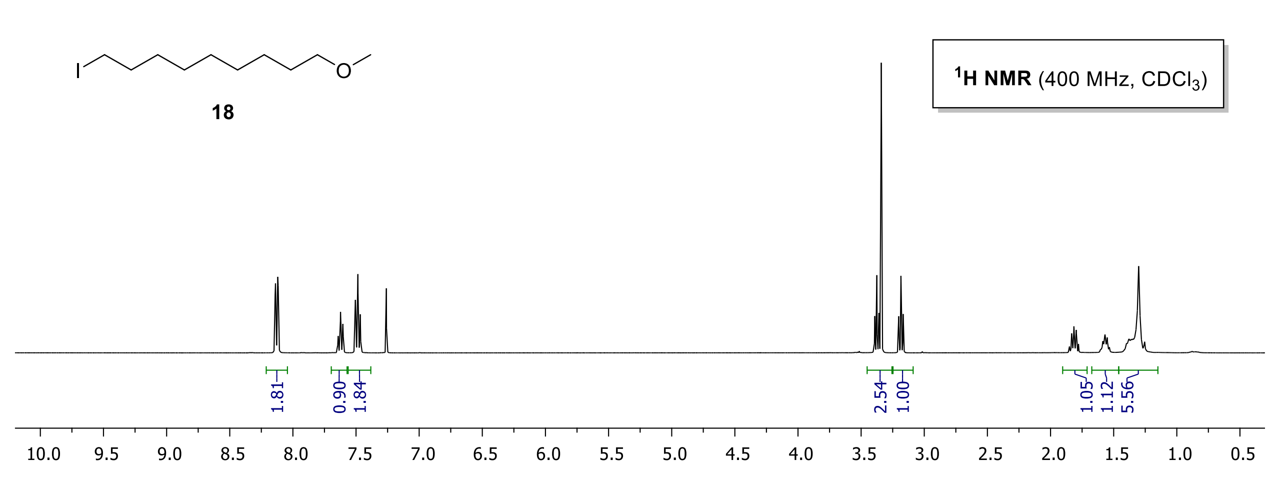

**Step 1**	m_s = 5.38 mg, m_{IC} = 4.06 mg, P_{IC} = 99.5%
**Step 2**	Int_t = 0.52,[a] n_t = 1
**Step 3**	Int_{IC} = 0.91,[b] n_{IC} = 1
**Step 4**	MW_t = 284.18 g/mol, MW_{IC} = 122.12 g/mol
**Step 5**	$P\,[\%] = \frac{n_{IC}\cdot Int_t\cdot MW_t\cdot m_{IC}}{n_t\cdot Int_{IC}\cdot MW_{IC}\cdot m_s}\,P_{IC} = \mathbf{99.8\%}$

[a] The integral of the target analyte was calculated as the average of signals at 1.47-1.65, 1.75-1.88, 3.18 and 3.30-3.40 ppm

[b] The integral of the Internal Calibrant was calculated as the average of signals at 7.45, 7.58 and 8.00 ppm

Biological Evaluation

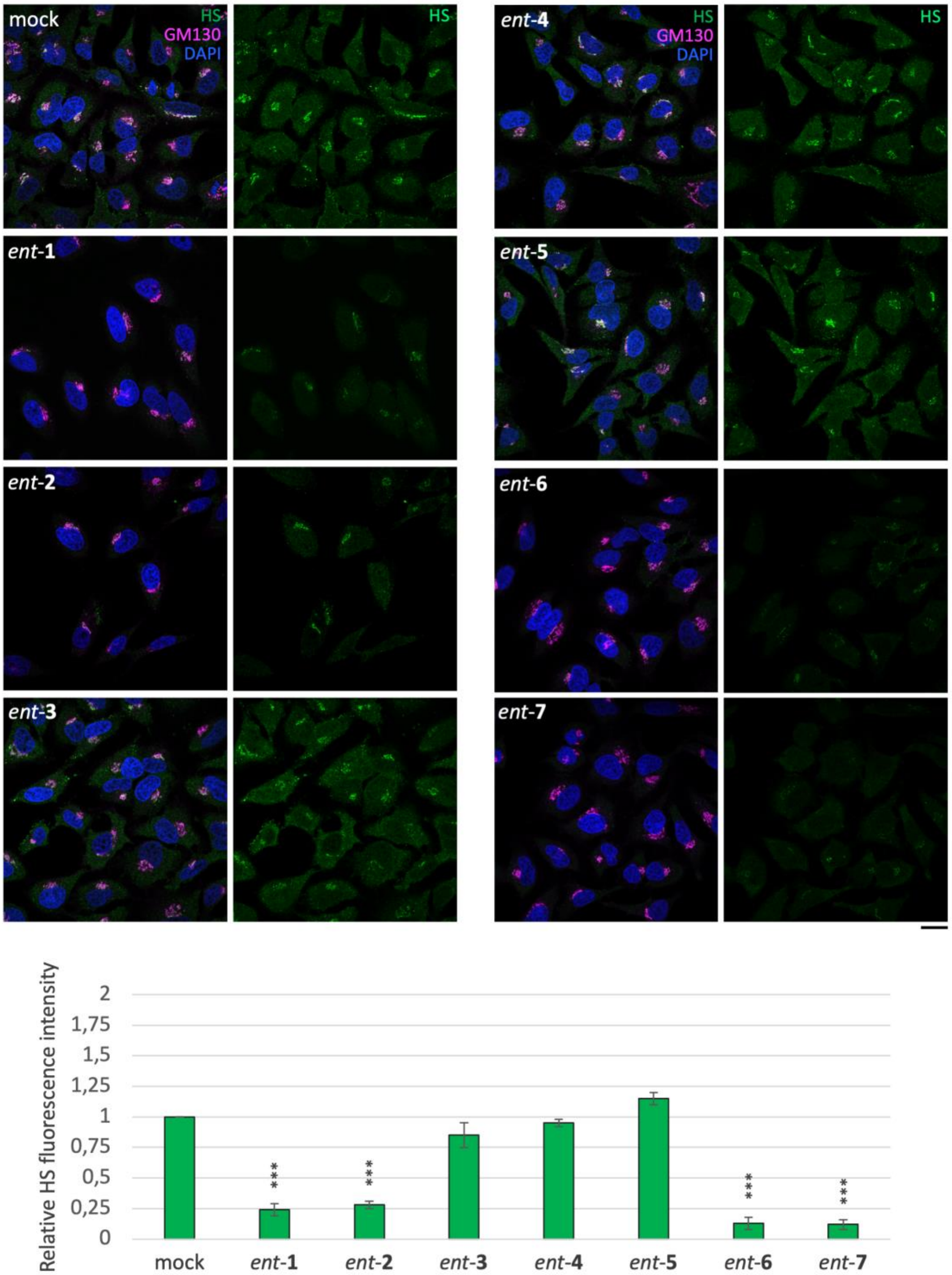

Figure S1. Heparan sulfate (HS) staining of the Hela cells treated with the L-iminosugars.

Hela cells seeded on coverslips were grown for 48 hours in the presence of 20 μM of each L-iminosugar, and then processed for indirect immunofluorescence by using specific antibodies against HS and GM130 (Golgi marker) and decorated with DAPI (Nuclear marker). Quantifications of immunofluorescence staining: the histograms represent the quantification based on the mean fluorescence signal of HS staining. 50 randomly chosen cells from three independent experiments were used for quantifications. Single focal sections are shown. Scale bar: 50 μm. Asterisks indicate the statistically significant differences: (***) p-value < 0.0001. Single focal sections are shown. Scale bar: 50 μm.

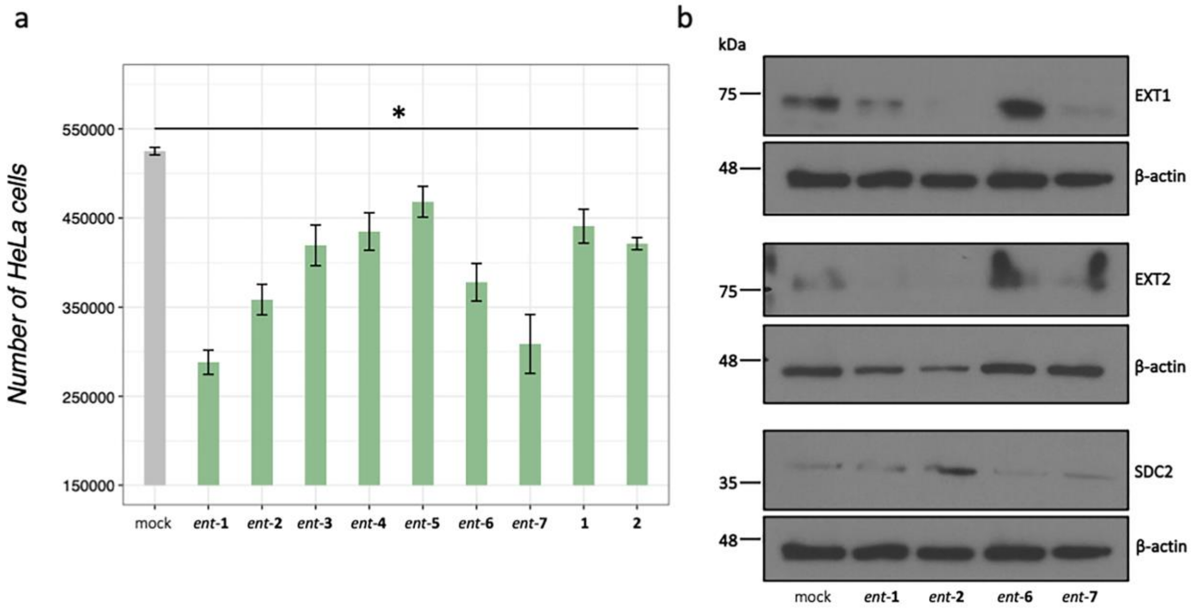

Figure S2. Effects of the iminosugars on HeLa cells proliferation and on EXT1, EXT2 and SDC2 protein levels.

(**a**) Cell count on HeLa cells treated with iminosugars. HeLa cells were exposed to iminosugars for 48 hours at a concentration of 20 µM. Untreated cells (mock) served as a control. The number of viable cells was determined by the trypan blue exclusion method. The data reported represent the mean values from three independent experiments performed in triplicate. Vertical bars indicate s. e. m. (*) p-value < 0.05.

(**b**) Western blotting analysis of EXT1, EXT2, SDC2 protein levels in Hela cells, untreated or treated with *ent*-1, *ent*-2, *ent*-6 and *ent*-7. To monitor equal loading of the proteins in the gel lanes, the blots were re-probed using an anti-β-actin antibody.
